# A simple model explains the cell cycle-dependent assembly of centromeric nucleosomes in holocentric species

**DOI:** 10.1093/nar/gkab648

**Published:** 2021-08-05

**Authors:** Amanda Souza Câmara, Veit Schubert, Martin Mascher, Andreas Houben

**Affiliations:** Leibniz Institute of Plant Genetics and Crop Plant Research (IPK) Gatersleben, 06466 Seeland, Germany; Leibniz Institute of Plant Genetics and Crop Plant Research (IPK) Gatersleben, 06466 Seeland, Germany; Leibniz Institute of Plant Genetics and Crop Plant Research (IPK) Gatersleben, 06466 Seeland, Germany; Leibniz Institute of Plant Genetics and Crop Plant Research (IPK) Gatersleben, 06466 Seeland, Germany

## Abstract

Centromeres are essential for chromosome movement. In independent taxa, species with holocentric chromosomes exist. In contrast to monocentric species, where no obvious dispersion of centromeres occurs during interphase, the organization of holocentromeres differs between condensed and decondensed chromosomes. During interphase, centromeres are dispersed into a large number of CENH3-positive nucleosome clusters in a number of holocentric species. With the onset of chromosome condensation, the centromeric nucleosomes join and form line-like holocentromeres. Using polymer simulations, we propose a mechanism relying on the interaction between centromeric nucleosomes and structural maintenance of chromosomes (SMC) proteins. Different sets of molecular dynamic simulations were evaluated by testing four parameters: (i) the concentration of Loop Extruders (LEs) corresponding to SMCs, (ii) the distribution and number of centromeric nucleosomes, (iii) the effect of centromeric nucleosomes on interacting LEs and (iv) the assembly of kinetochores bound to centromeric nucleosomes. We observed the formation of a line-like holocentromere, due to the aggregation of the centromeric nucleosomes when the chromosome was compacted into loops. A groove-like holocentromere structure formed after a kinetochore complex was simulated along the centromeric line. Similar mechanisms may also organize a monocentric chromosome constriction, and its regulation may cause different centromere types during evolution.

## INTRODUCTION

Centromeres are required for the movement of chromosomes during cell division. Most organisms contain a single size-restricted centromere per chromosome (monocentromere), visible as a primary constriction during metaphase. However, in independent eukaryotic taxa, including some protists, plants and invertebrates, species with holocentric chromosomes have evolved repeatedly (1–3). Holocentric chromosomes have no distinct primary constriction visible at metaphase. Instead, spindle fibres are attached along almost the entire poleward surface of the chromatids (4). Due to the chromosome-wide distribution of holocentromeres, single-chromatids cohere along the entire chromatids and appear as two parallel structures at metaphase. In contrast, in monocentric species, the sister chromatid cohesion is restricted to a single position at the centromere, and X-shaped metaphase chromosomes are formed.

Clades possessing holocentromere types include >350 000 species in total (5). Likely, holocentricity is even more common than reported so far as the identification of the centromere type is challenging for small chromosomes. One common explanation for the development of holocentric chromosomes during evolution is their putative advantage to tolerate DNA double-strand breakages inducing chromosomal fragments, which will not be lost during cell division (6).

Different types of holocentromeres exist, as exemplified by either presence or absence of the centromere-specific histone H3 variant CENH3 (also called CENPA) and centromere-specific repeats, different morphology of chromosomes and diversity of meiotic behaviour (4,7,8). The mechanism that gives rise to holocentricity is still unknown. However, the fact that holocentrics arose independently several times during evolution suggests that the transition from mono- to a holocentromere type may be a relatively simple process.

**Figure 1.**
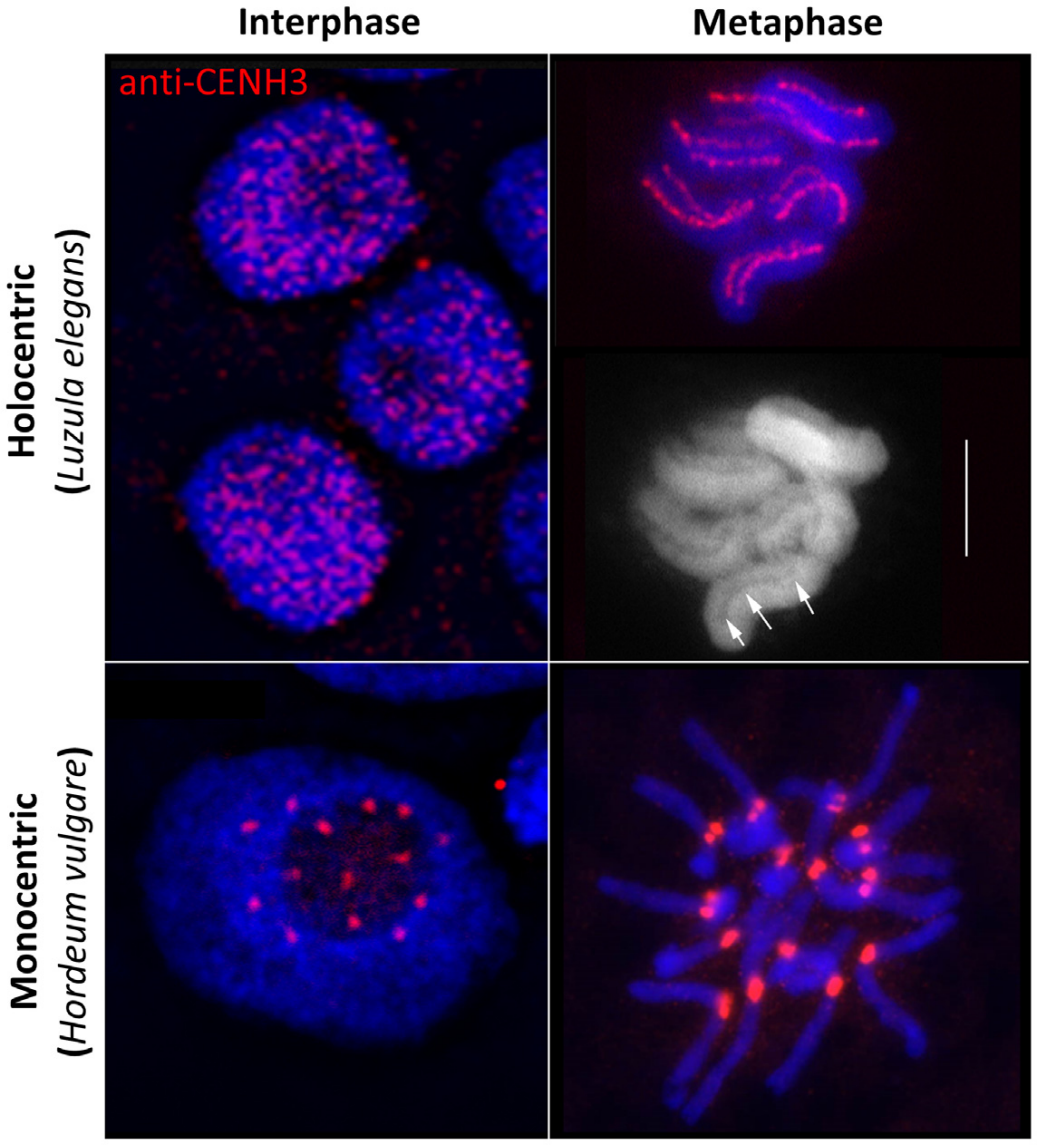
Interphase and metaphase chromosomes of holocentric (*Luzula elegans*) and monocentric (*Hordeum vulgare*) species. During interphase, e.g. in *L. elegans*, holocentromeres disperse into a large number of CENH3-positive centromeric nucleosome clusters. With the onset of chromosome condensation, the centromeric nucleosome clusters join and form line-like structures along both chromatids. In contrast, in most monocentric species, e.g. in *H. vulgare* no obvious dispersion of the centromeres occurs during interphase. CENH3 (red) indicates the position of centromeres. Arrows indicate the longitudinal centromere groove in *L. elegans*. Chromosomes were counterstained with DAPI; bar = 5 μm. Pictures were taken from Heckmann *et al.* (11) with permission from S. Karger A.G., Basel.

In contrast to most monocentric species, where no obvious dispersion of the centromeres occurs during interphase, the organization of holocentromeres differs between interphase and mitotic metaphase (Figure 1). During interphase, e.g. in the nematode *Caenorhabditis elegans* (9) and plant species, the Juncaceae *Luzula elegans* (10,11) and the Cyperaceae *Rhynchospora pubera* (12) holocentromeres are dispersed into a large number of CENH3-positive centromeric nucleosome clusters, which are evenly distributed within the nucleus. With the onset of chromosome condensation, the centromeric nucleosome clusters join and form line-like structures along both chromatids. After segregation of chromatids, dispersion of holocentromeres is concomitant with chromatin decondensation. Hence, the organization of holocentromeres is cell cyle-dependent and the line-like metaphase centromere is a result of fused centromeric nucleosomes. Due to this multi-centromere subunit structure, holocentric chromosomes could also be considered as ‘polycentric’ as proposed by (13). However, also a monocentromere is composed of multiple centromeric nucleosomes based on the centromere subunit model, where the centromere is assembled from repetitive subunits tandemly arranged on a continuous chromatin fibre (14). An additional centromere feature of the holocentric genera *Luzula* and *Rhynchospora* is the presence of a longitudinal groove along the poleward surface of mitotic metaphase chromosomes (Figure 1) (10–12,15). However, this centromere structure does not exist in all holocentric species (12,16).

The peculiar cell cycle dynamics and structure of holocentromeres prompt us to apply a loop extrusion model to decipher the potential mechanism behind the cell cycle-dependent assembly of centromeric nucleosomes and the formation of a groove-like centromere structure. It has been shown that the extrusion of chromatin loops affects the condensation and segregation of sister chromatids (17). Loop extrusion relies on the action of structural maintenance of chromosomes (SMC) complexes. SMC proteins are a group of evolutionarily conserved protein complexes including cohesins, condensins and SMC5/6 complexes sharing similar structures and dynamics (18,19). They bind to the DNA molecule and translocate along it, progressively bringing together loci separated by larger distances in the chromosome and leaving a DNA loop behind (20–22). But neither do we know the exact mode of action of SMC complexes nor is it clear whether differences exist between species (18).

In interphase, the motion of SMC complexes can be affected by other proteins such as the CCCTC-binding factor (CTCF) or the wings apart-like protein (WAPL), which were reported to anchor or release the approaching SMC complexes (18). By anchoring, for example, CTCFs are proposed to fix loop bases, thus delimiting regions highly self-contacting, called topological associating domains (TADs) (23). SMC complexes themselves can also block each other, leading to the formation of more compact loop arrangements (24). With the onset of cell division, SMC complex concentration varies (25) and generates different chromatin condensation levels.

In mitotic chromosomes, cohesins are related to sister chromatid cohesion, while the condensation of chromosomes relies on the function of condensin I and II (26,27). In vertebrates, the two condensins have been associated with different phases of the cell cycle. Condensin I becomes active after nuclear membrane breakdown, whereas condensin II is active already in G2 (25,28).

In monocentric species, condensin II acts on the axial shortening of the chromosome, while condensin I acts on the lateral compaction (26). Originally, condensin I was believed to be lost and not required in holocentric species (25) until its later discovery in *C. elegans* (29). Nonetheless, the condensation of mitotic chromosomes is mainly attributed to condensin II, whose depletion profoundly affects prophase condensation. Condensin II is present along the holocentromeres of mitotic *C. elegans* chromosomes, but condensin I appears dispersed, and its depletion did not cause prophase condensation defects (29). The occurrence of SMC complexes is possibly a general feature of holocentric chromosomes, as the cohesin α-kleisin subunit also colocalizes with the holocentromere of the plant *L. elegans* (30).

In this work, we simulated the cell cycle-dependent formation of a holocentromere-like chromosome based on the condensation of a single chromatin fibre possessing a large number of centromeric nucleosomes. To keep our simulation as simple as possible, we considered only the compaction by a general SMC complex type, what we called Loop Extruder (LE). Additionally, we applied possible chromatin fibre crossings, mimicking the action of topoisomerase II, as described in (24). Other factors involved in the process of chromosome condensation were ignored. The LE worked as postulated in the loop extrusion model of (31). When the extruded loops grow larger, more distant DNA regions can interact, allowing chromatin domains to be compacted by loops. We assumed a scattered distribution of centromeric nucleosomes, proposing they affect the LEs motion. Like CTCF and WAPL proteins, centromeric nucleosomes were already suggested to affect the loop extrusion process because the compaction of chromatin is interrupted at centromeres with chromatin loops limited to the pericentromeric region (32,33).

We performed different sets of molecular dynamic simulations by testing four parameters: (i) the concentration of LEs, (ii) the distribution and number of centromeric nucleosomes, (iii) the effect of centromeric nucleosomes on interacting LEs and (iv) the assembly of kinetochores bound to centromeric nucleosomes. We observed the formation of a line-like holocentromere, due to the aggregation of the centromeric nucleosomes when the chromosome was compacted into loops. A groove-like holocentromere structure was formed after a kinetochore complex was simulated along the centromeric line. Simulation of a monocentric chromosome suggests that similar mechanisms may also organize a monocentric chromosome constriction.

## MATERIALS AND METHODS

All simulations represent single ∼20 Mb-long chromosomes, modelled as a polymer chain with 100 000 monomers. Each monomer corresponded to one nucleosome. Centromeric nucleosomes are uniformly distributed along the chromosome. They differed from noncentromeric nucleosomes only regarding the interaction with the simulated LEs. The LE was simulated as a dynamic bond between two nucleosomes.

### One-dimensional (1D) simulations of loop extrusion

We performed 1D simulations to determine which nucleosomes the LEs bind as a function of time. ln the simulation, the LEs initially bound pairs of adjacent nucleosomes. There was always the same number of LEs during the simulation. The nucleosome pairs that LEs bind changed according to the extrusion motion and the interaction rules.

For the extrusion motion, we applied the algorithm of Alipour and Marko (31) and Goloborodko *et al.* (24). We adopted two-sided LEs because (34) showed that one-sided LEs could not reproduce alone some biological phenomena. As a two-sided extrusion motion, both nucleosomes bound by a LE progressively changed. The left-side nucleosome always changed to the one on its left and the right-side nucleosome to the one on its right. This change occurred once every 1D step, which is the velocity of the LE. With this extrusion motion, the LE bound progressively more distant nucleosomes, until the LE unbound and reinitiated its motion at another side, with a new pair of nucleosomes. This occurred with a chance of one over 1000 1D steps, a period called lifetime.

Two interaction rules affect the extrusion. One nucleosome cannot be bound by more than one LE, and adjacent LEs block each other’s way but can continue the extrusion in the opposite direction, i.e. that not occupied by another LE. When a LE meets a centromeric nucleosome one of the three settings apply: (i) no effect—the LE continues its motion freely; (2) blocking effect—the LE motion is blocked on one side and (3) anchoring effect—the LE is blocked and not allowed to unbind, even past its lifetime. In the last two settings, the centromeric nucleosomes were not allowed to be occupied by a LE.

The final list of nucleosomes bound by LEs overtime was later used to (i) calculate the chromosome and average chromatin loop lengths, (ii) verify if the simulation has reached a compaction equilibrium, (iii) analyse the localization of LEs after compaction and (iii) pass the data over to three-dimensional simulations.

### Lengths of chromosome and average chromatin loop in number of nucleosomes

**Figure 2.**
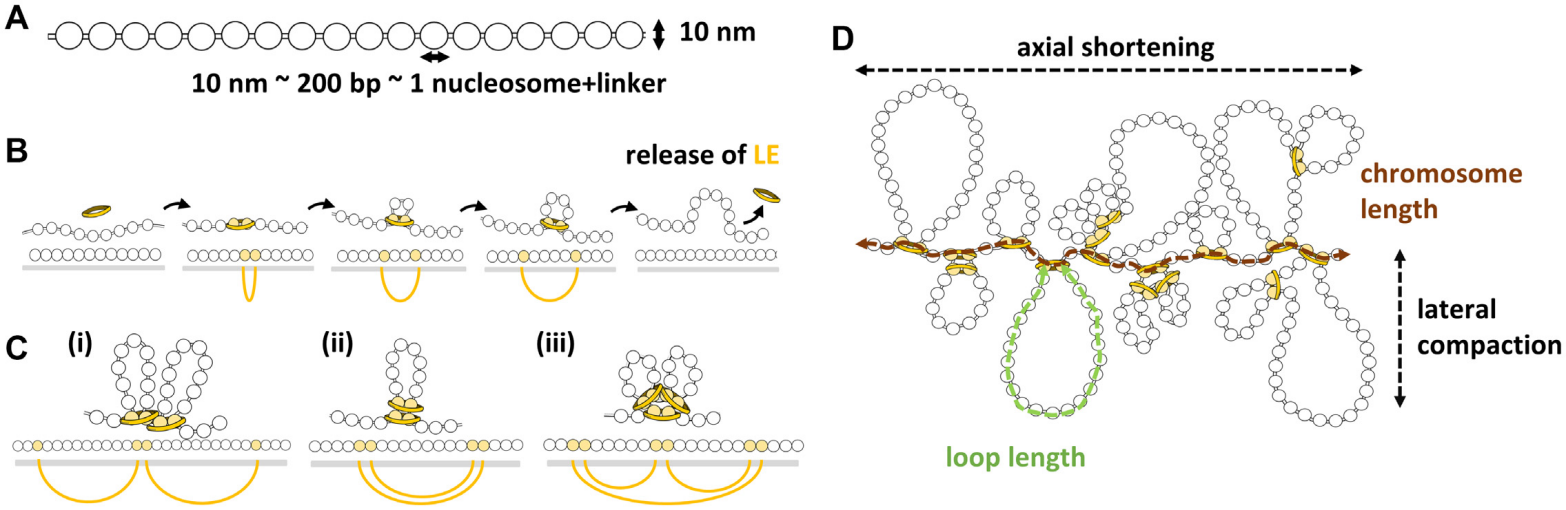
Schematic representation of the adopted chromosome and loop extrusion models. (**A**) The chromosomal 10 nm chromatin fibre represented as a beads-on-a-string polymer. Around ∼200 base pairs of DNA (including the linker) are wrapped around each nucleosome. (**B**) The loop extrusion model. The Loop Extruder (LE) is represented by a yellow ring. Nucleosomes bound by LEs are shown in yellow, the bond between them is represented as a yellow ellipsoid line, and the grey bar represents the chromatin. (**C**) Different examples of loops formed by two proximal LEs: (i) side-by-side loops, (ii) nested loops and (iii) combination of both. (**D**) Chromosome condensation by loop formation. The bases of the loops form the axis of the chromosome, and the loops are radially distributed. The degree of chromosome condensation is due to axial shortening and lateral compaction. These two parameters are functions of the number of nucleosomes and can be computed as the chromosome length and the average loop length, respectively. In this example, the chromosome length is 18 axial nucleosomes and the loop length is 23 nucleosomes. We define axial nucleosomes as bound by LEs and outside any loop.

We calculated the chromosome length as the total number of nucleosomes forming the basis of loops—coinciding with nucleosomes bound by LEs—but outside other loops. For example, if there were no nested loops, as in a sparse state, the chromosome length is simply the total number of nucleosomes bound by LEs (twice the number of LEs). This definition does not describe sparse states well, because in the deconsensd state, the length of a chromosome is not a well-defined concept. But for compacted states this definition appears proportional to experimental data (Supplementary Tables S1 and S2). In the compacted state, these nucleosomes form the basis of consecutive (side-by-side) loops, which may be nested or not (Figure 2). We name the nucleosomes bound by LEs and outside loops the ‘axial nucleosomes’. The average chromatin loop length was calculated as the sum of nucleosomes between each pair of LE-bound nucleosomes over the number of LEs. We considered that the simulation reached an equilibrium when these two parameters were stable over time. To verify this, we performed 10 replicates of each model.

### Three-dimensional (3D) polymer simulations of single chromosomes

We performed Langevin dynamics simulations with OpenMM Python API (Application Programming Interface) (35), using the integrator VariableLangevinIntegrator with 300 K temperature, 0.001 ps^–1^ friction coefficient and 80 fs error tolerance. We used the python library from https://github.com/mirnylab/ to implement simulation parameters. The following three forces composed the force field of the chromosome: (i) harmonic force between adjacent nucleosomes, with 10 nm mean distance between them and 1 nm wiggle distance—this conferred the chromatin fibre of 10 nm thickness; (ii) Grosberg stiffness force with 1 *k*_B_*T* stiffness constant and (iii) repulsive polynomial force up to 10.5 nm distance, that allowed crossing of the chromatin fibre over 5 *k*_B_*T* energy—mimicking the presence of topoisomerase II, as in (36). This force field was valid to all centromeric and non-centromeric nucleosomes.

The list of nucleosomes bound by LEs over time (retrieved from the 1D simulation) integrated the loop extrusion into the 3D simulations. The 1D simulation accounted for the effects of the centromeric nucleosomes in the loop extrusion, and this was enough to distinguish them from the other nucleosomes. The binding of nucleosomes by LE was simulated as a harmonic force with 5 nm mean distance and 0.5 *k*_B_*T*/nm^2^ harmonic force constant, as in (32). This force was updated every block of 3D steps (100 3D steps), according to the bonds list from the 1D simulation, where a 1D step corresponds to a block of 3D steps. The simulation started with a random conformation, ran the first 10 000 blocks without loop extrusion and then ran 40 000 more blocks with a constant number of LEs.

### 3D simulations with a kinetochore

This simulation presented two different objects to which different forces were applied, chromatin and the kinetochore. Chromatin was simulated as above. The kinetochore was simulated by fixing non-connected beads on a regular grid with length proportional to the number of centromeric nucleosomes (Supplementary Table S3). Only two forces acted upon the kinetochore beads: a tethering harmonic force with 5 *k*_B_*T*/nm^2^ spring constant and a polynomial repulsive force as before, but with 10 000 *k*_B_*T* energy truncation, so the chromatin fibre could not cross the kinetochore. Centromeric nucleosomes were also tethered during the simulation. In the initial conformation, the centromeric nucleosomes were aligned along the *z*-axis, and non-centromeric nucleosomes formed straight chromatin loops along the *x*-axis. In the monocentric model, axial nucleosomes outside the centromere region were also initially placed along the *z*-axis. The data for these loops were taken from 1D simulations of loop extrusion. The 1D simulation ran for 100 000 1D steps, but only the last 50 000 were used in the 3D simulations with the kinetochore. This allowed the system to equilibrate while still performing loop extrusion. Supplementary Table S3 gives a summary of simulation parameters along with their justifications.

### Analysis of 3D simulations

To evaluate the space occupied by centromeric nucleosomes, we calculated the spatial distance between centromeric nucleosomes adjacent in the linear genome and built histograms.

To evaluate the chromosome length, we calculated the median and peak distances between sequence-consecutive axial nucleosomes. The peak distance is the most populated distance in a histogram binned at 0.5 nm. These representative distances were calculated from 10 conformations over the last 10 000 block of 3D steps. The 3D chromosome length was then calculated as the number of axial nucleosomes in the last conformation times the median or peak distance between them. This value is verified with manual measurements of consecutive segments of LEs visually identified using pymol (Supplementary Figure S1) (37).

Contact matrices were built using 50 simulations (5 000 000 steps long) and 10 conformations of each equally separated over the last 100 000 steps. We show matrices of a 4 Mb region, with uniform bins of 4 kb (20 nucleosomes). A contact is considered when 2 nucleosomes are at least 200 nm apart (38). For the contact probability, we used only the final conformation of a single simulation. We considered a range of 10 kb to 20 Mb chromosomic distances, binned on a logarithmic scale. The contact probability for each bin was calculated as the sum of all observed contacts within the distance range of this bin and divided by the number of all possible pairs of nucleosomes within the same distance range.

## RESULTS

### Prerequisites of the model

We simulated the cell cycle-dependent condensation process of a modelled holocentric chromosome to test factors involved in the line-like assembly of centromeric nucleosomes during mitosis. All simulations represented a ∼20 Mbp-long chromosome, corresponding to ∼100 000 nucleosomes. Chromosomes of this length exist in the holocentric worm *C. elegans* (39).

Our dynamic model was based on the following assumptions. The chromosomal 10 nm chromatin fibre is represented as a beads-on-a-string polymer (Figure 2A), in which each bead corresponds to one nucleosome containing two copies of histone H3, H4, H2A and H2B. We assumed that ∼ 200 bps of DNA represent 147 bps wrapped around each nucleosome plus the nucleosome linker DNA (24). Ring-like SMCs were simulated as chromatin fibre LEs. They progressively bind distant nucleosomes but can also unbind from the chromatin fibre (Figure 2B). When two LEs meet during the chromatin loop extrusion process, they block each other’s way, as proposed by Alipour and Marko (24,31), thus generating side-by-side and nested loop arrangements, as shown in Figure 2C. Dynamic binding and release of LEs from the chromatin fibre resulted in a compacted mitotic chromosome, in agreement with the simulations of Goloborodko *et al.* (24).

The degree of chromosome condensation can be determined by two parameters: lateral compaction and axial shortening of chromosomes (26). We associated these two parameters to the average chromatin loop and chromosome lengths, respectively (Figure 2D). We defined the chromosome length as the total number of nucleosomes that are the basis of the loops but are at the same time outside other loops, which we named ‘axial nucleosomes’. We defined the loop length as the number of nucleosomes inside the loops. These parameters can be measured regardless of the presence of centromeric or non-centromeric nucleosome. They reached an equilibrium in the later stages of the simulation (Supplementary Figure S2), which may be a sparse or a compacted state. We used both parameters and the percentage of nucldeosomes outside chromatin loops to compare the degree of chromosome compaction of final conformations obtained after different simulations.

The dynamic behaviour of centromeric nucleosomes is an integral part of our model. We randomly chose the positions of centromeric nucleosomes, uniformly distributed, to mimic holocentric or monocentric chromosomes. The only unique feature of centromeric nucleosomes was their ability to interfere with the motion of LEs.

The release of a LE was determined by its lifetime, a computational parameter that relates to the experimental affinity of condensins to the DNA. But the interaction with centromeric nucleosomes might alter the dynamics of the LEs (32). Other proteins were already found to interfere with the motion of SMCs. CTCFs, for example, anchor cohesins and prevent their release (40). WAPL releases cohesin and prevents the extension of chromatin loops (41).

**Figure 3.**
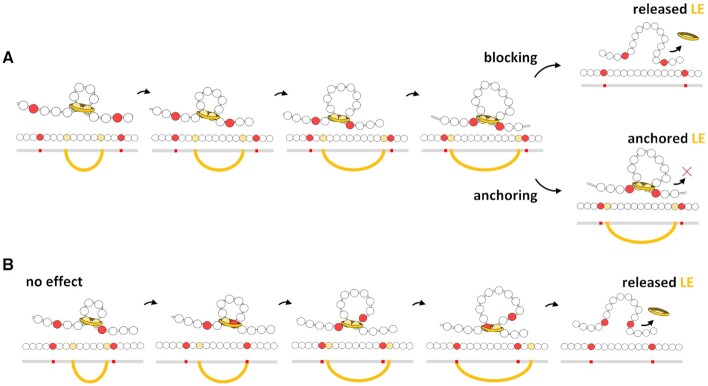
Effects of the presence of centromeric nucleosomes (in red) in the loop extrusion process. (**A**) Blocking and anchoring effects. In both, one centromeric nucleosome blocks the motion in its direction, but the opposite side of the LE (in yellow) continues to reel. In the blocking effect the LE interacting with the centromeric nucleosome can unbind, but in the anchoring effect the LE is permanently bound to the centromeric nucleosome. (**B**) For comparison, centromeric nucleosomes that do not interact with the LE can pass through it.

We considered two different interaction effects of the centromeric nucleosomes (Figure 3) and compared them to a lack of effect, which allowed LEs to pass freely, as at non-centromeric nucleosomes. As a barrier of LEs, a centromeric nucleosome partially blocks the motion of the LE. As shown in Figure 3A, when a LE meets a centromeric nucleosome, the motion of the LE in the direction towards the centromeric nucleosome is blocked. In the opposite direction, the LE continues to reel and to extrude a loop until the eventual LE release. As an anchor of LEs, a centromeric nucleosome partially blocks the motion and prevents the release of the LE. When a LE meets a centromeric nucleosome, it continues to reel in the opposite direction until meeting another LE or centromeric nucleosome (Figure 3A). For comparison, Figure 3B exemplifies a loop extrusion by one LE if centromeric nucleosomes would have no effect in its motion.

Besides, we considered the cell‐cycle dependent, centromeric localization of kinetochore proteins. The kinetochore is a multiprotein complex that connects centromeric nucleosomes to the microtubules and is considerably larger than a nucleosome. The composition of the kinetochore is cell cycle-dependent and forms, at both metaphase chromatids, a plate-like structure at the side of centromeric nucleosome clusters (1,42,43). Thus, we simulated an orderly arrangement of kinetochore units, as a set of beads with fixed positions in a grid forming a chromosome-wide, plate-like structure. Then, we tethered the centromeric nucleosomes to one side of this kinetochore arrangement, leaving the opposite side free to interact with microtubules, although microtubules were not simulated.

Table 1 lists the simulation parameters we varied in this work and the three hypotheses on the arrangement of centromeric nucleosomes.

**Table 1.** Main features of the proposed hypothesis and tested parameters for the modelled 20 Mb-sized chromosome

Hypothesis on	Number of LEs	Centromere type	Interaction effects of centromeric nucleosomes	Kinetochore structure
***Formation of the centromeric line***	50–2000	Holocentromere	None, blocking, or anchoring	Not considered
***Different centromere types***	1000	Mono- and holocentromere	Blocking or anchoring	Not considered
***Holocentric groove***	1000	Mono- and holocentromere	Anchoring	Plate-like grid of beads

### Anchoring of Loop Extruders (LEs) by dispersed centromeric nucleosomes leads to the formation of a line-like holocentromere during chromosome condensation

**Figure 4.**
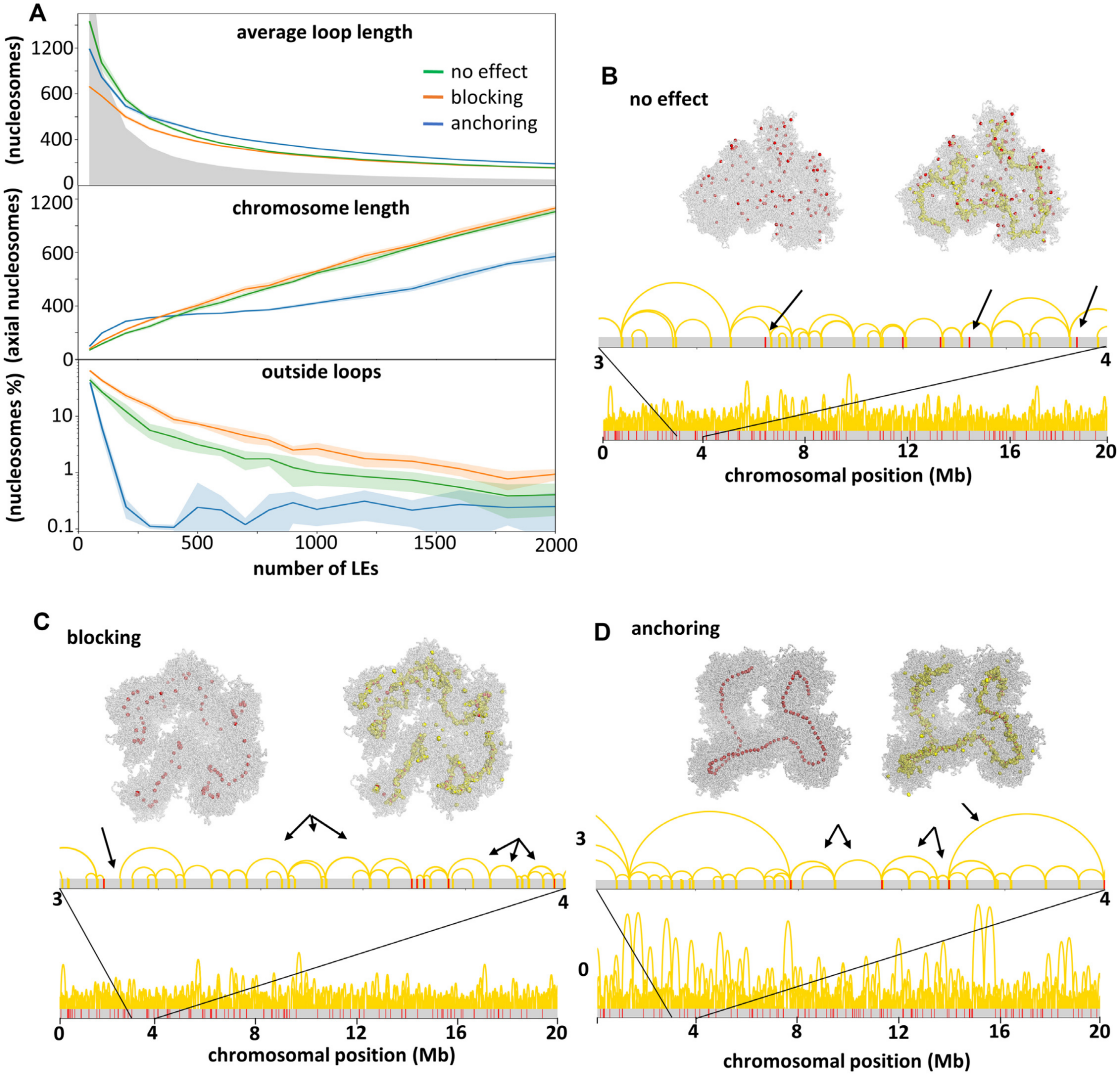
Effects of centromeric units. (**A**) Chromosome length and average loop length as a function of the number of simulated LEs, and the number of nudeosomes outside chromatin loops. These parameters were calculated from simulations considering three different effects (no effect, blocking or anchoring) of centromeric units in the loop extrusion process. The grey area characterizes sparse states, when the number of LEs times the average loop length is <100 000 nucleosome. Above the grey area the chromosome is in a compacted state, with nested chromatin loops. (**B**–**D**) Final conformations of three simulations (100 000 3D steps long) with different centromeric effects (see Supplementary Movies S1–3 for simulation examples). The distribution of centromeric nucleosomes (red) and LEs (yellow) is shown in the chromatin fibre (grey), in the 3D structure (top) and sequence (bottom). For each conformation arrows indicate characteristic loop organizations. (B) With no effect, loops are observed spanning centromeric nucleosomes. (C) With the blocking effect, regions are observed outside loops as well as multiple loops between two adjacent centromeric nucleosomes. (D) With the anchoring effect, only one or two loops are observed between adjacent centromeric nucleosomes.

We tested sixteen different amounts of LEs (between 50 and 2000) interacting with a single 20 Mb-long chromatin fibre, containing 100 uniformly distributed centromeric nucleosomes. A comparable distribution exists in *C. elegans* (44). We quantified the degree of chromosome condensation relative to the amount of LEs by calculating the average chromatin loop and chromosome lengths (Figure 4A). A sparse state is easily identified when the existing loops cannot cover the extent of the chromosome, marked by the grey area in the graphic (when the average chromatin loop length times the number of LEs is <100 000 nucleosomes). Above the grey area the LEs accumulate in the chromosome axis and the chromatin loops become nested, characterizing a compacted state.

We first considered the setting where centromeric nucleosomes make no effect on the loop extrusion process (Supplementary Movie S1). We observed that the presence of LEs alone (above 100 units) could bring the chromosome to a condensed state, as reported in (24,31). The more LEs were considered, the more condensed was the chromosome; the average loop length and the percentage of nucleosomes outside chromatin loops decreased. The damped increase in the chromosome length indicates that the LEs are rather accumulating in nested loops than creating new adjacent loops. But after compaction, centromeric nucleosomes were dispersed and not arranged in a holocentromere-like manner (Figure 4B), and LEs localize uniformly along the chromosome (Supplementary Figure S3).

Then, we considered that centromeric nucleosomes block the motion of LEs (Supplementary Movie S2). Again, an increase in the number of LEs repeated the pattern for chromosome and average loop lengths seen with the no effect model, bringing the chromosome into a more condensed state (Figure 4C). Centromeric nucleosomes were arranged in small groups, preferentially central to the chromosome axis. These centromeric nucleosomes were brought together by the blocking effect of the LEs, becoming a focal point of LE accumulation (Supplementary Figure S3). This grouping of centromeric nucleosomes was only transient, since LEs could be released. In contrast to the model where centromeric nucleosomes had no effect on LE, we always observed the centromeric nucleosomes outside or at the boundaries of chromatin loops. The higher percentage of regions free of loops is in accordance with the slightly larger chromosome length and shorter average loop length.

Last, we considered that centromeric nucleosomes anchored LEs, i.e. prevented them from unbinding from the chromatin (Supplementary Movie S3). The length of the chromatin loops again decreased with the increase of the LEs number. But with 200 LEs, the chromosome length reached a plateau of <400 nucleosomes (Figure 4A). This number corresponds to 200 chromatin loops in between 100 centromeric nucleosomes. These loops were anchored by LEs and organized side-by-side (Figure 4D, arrowed). Due to the persistent LE anchoring, the proximity of the centromeric nucleosomes became permanent instead of transient. At the end of the condensation process, the entire chromosome was folded into chromatin loops (the centromeric nucleosomes account for the 0.1% of nucleosomes outside loops). Inbetween two centromeric nucleosomes, there were always one or two chromatin loops, and colocalization of LEs and centromeric nucleosomes is higher than in the blocking case (Supplementary Figure S3). With the anchoring effect of centromeric nucleosomes, it was possible to model a holocentromere-like structure formed by side-by-side centromeric nucleosomes. In the simulations considering the anchoring effect, the chromosome length remained approximately the same with 200–1000 LEs. 200 LEs were enough to anchor all (100) centromeric nucleosomes into a line. The remaining LEs accumulated around the holocentromere-like line and further divided the anchored chromatin loops into smaller loops, leading to a lateral compaction of the chromosome. More than 1000 LEs again increased the chromosome length, and the centromeric nucleosomes were more distant to each other. This indicates that many LEs may hinder the formation of a holocentromere-like organization by the accumulation of LEs between centromeric nucleosomes.

Simulations twice as long showed that the chromosome length tends to be <400 axial nucleosomes even with a large number of LEs for the anchoring effect; i.e. if there were infinite time, the centromeric nucleosomes would always come to a compact line, even with a high concentration of LEs. But the longer time did not change the chromosome or loop lengths in any case for the other settings (no or blocking effect) (Supplementary Figure S4).

**Figure 5.**
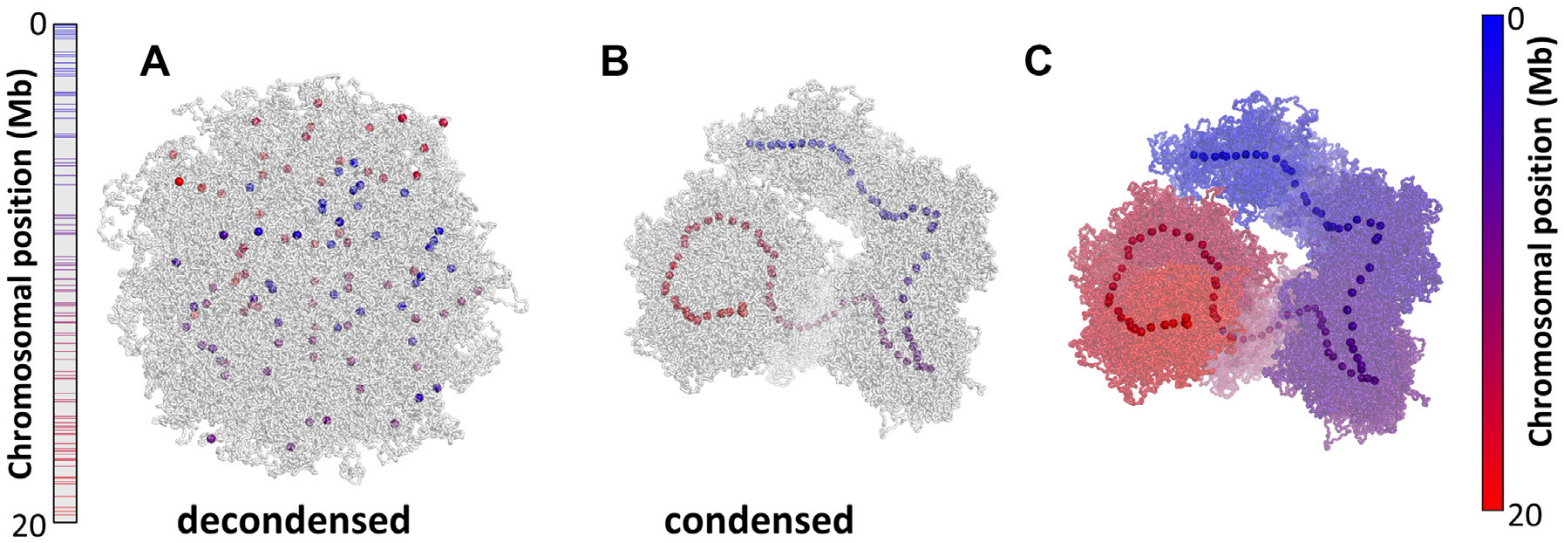
Simulated conformations of a holocentric-like chromosome with 100 centromeric nucleosomes with anchoring effect after condensation by 1000 LEs (see Supplementary Movie S3 for the entire simulation). Centromeric nucleosomes are colored from blue to red according to the position in the linear genome. Left bar represents the chromosome length and colored lines indicate the position of centromeric nucleosomes. (**A**) The decondensed conformation represents an interphase with dispersed centromeric nucleosomes. (**B**) Condensation of a chromosome due to loop extrusion. Centromeric nucleosomes are aligned in the axis of the chromosome following the position in the chromosomal sequence. (**C**) Condensed holocentric chromosome colored from blue to red, as indicated by the bar at the right. The chromosome is entirely linearly arranged along the chromosome axis, following the position in the chromosomal sequence.

When a holocentromere-like structure was formed, the centromeric nucleosomes were linearly organized, meaning that their position along the line followed their position along the chromatin fibre (Figure 5). The chromatin between centromeric nucleosomes was arranged into loops, whose bases were held together by LEs. Chromatin loops were also linearly organized, as expected for mitotic chromosomes (45). Compared to the anchoring of LEs, the other settings increased the chromosome length by >50% (Supplementary Table S2), with similar chromatin loop lengths (Figure 4A). We conclude that the centromeric nucleosomes modulate the length of the condensed chromosome. This result is consistent with the report by Maddox *et al.* (46), who observed an unusual condensation of chromosomes by depleting centromeric nucleosomes in *C. elegans*.

We verified the structural consequences of the three settings—no effect on, blocking or anchoring LEs—with three different analyses. First, manual measurements of the chromosome length confirm its relation to the number of axial nucleosomes and the shorter chromosome in the anchoring effect model (Supplementary Table S2 and Supplementary Figure S1). Second, histograms of the distance between consecutive centromeric nucleosomes showed that they were closer in the anchoring effect model (Supplementary Figure S5). Third, contact matrices and probability showed that larger distances could be brought into contact by anchoring of the LEs (Supplementary Figure S6). In this case, the regions between consecutive centromeric nucleosomes were enriched in internal contacts (even more at the borders), appearing as squares (with clear lines and corners) that extend beyond the main diagonal in the contact matrix. In the no effect case, the main diagonal is uniform along the chromosome. And in the blocking case, intermediate features appeared: contacts were enriched at the centromeric nucleosomes and lines marking squares in the contact matrix appear, but large squares lack the corners and do not extend beyond the main diagonal.

Especially for the blocking case, we considered if it could reproduce the anchoring effect with a longer lifetime, i.e. remaining for longer time bound at the chromatin. We changed the lifetime from 100 to 5000 3D steps and kept 1000 LEs (Supplementary Figure S7). But this could not bring the centromeric nucleosomes closer into a line, since many LEs were in between them, with slower dynamics. Longer lifetime approximated the chromosome and average chromatin loop lengths to the values with anchoring case. For 5000 steps lifetime, the chromosome length was significantly shortened. But for all tested lifetimes, the blocking effect still left regions without loops, preventing the formation of a holocentric line (Supplementary Figure S7).

### Clustered distribution of centromeric nucleosomes induces a monocentromere-like structure

We modelled the structure of holo- and monocentric chromosomes by changing the distribution and number of centromeric nucleosomes (Supplementary Movies S3 and 4). In the holocentric, 100 centromeric nucleosomes were normally distributed over the entire chromosome, and in the monocentric, 20 centromeric nucleosomes were clustered in a small region corresponding to 400 kb. In both cases, we simulated a ∼20 Mb long chromosome containing 1000 LEs.

**Figure 6.**
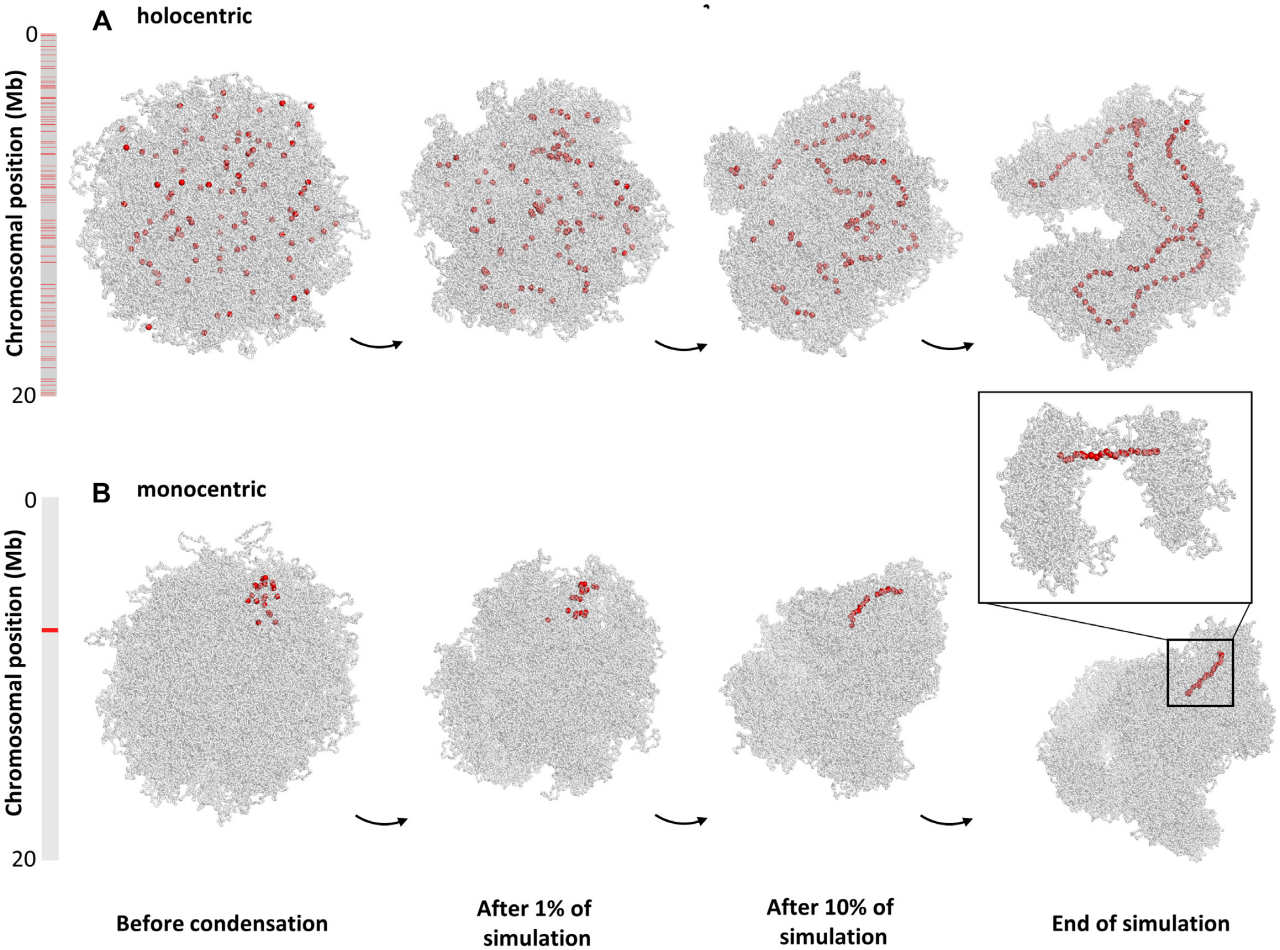
Comparison between simulated (**A**) holocentric- and (**B**) monocentric-like chromosomes at different stages of the condensation process (Supplementary Videos S3 and 4). Bars indicate chromosome length and red lines the position of the centromeric nucleosomes. (A) The condensed holocentric-like chromosome presents an average loop size of 325 nucleosomes and a chromosome length of 374 nucleosomes. (B) The condensed monocentric-like chromosome presents loop sizes of 59 and 260 nucleosomes inside and outside the centromeric region, respectively, and a chromosome length of 650 nucleosomes. The inset shows the centromeric region, with smaller chromatin loops, resembling the centromere constriction of monocentric chromosomes.

Non-compacted (interphase-like) and compacted (prophase-like) chromosome conformations for both centromere types were simulated (Figure 6). We only considered the prophase stage because highly compacted mitotic chromosomes arise from more than one condensation step (36,46). The non-compacted holocentric chromosome presented abundant and scattered centromeric nucleosomes. In contrast, the centromeric nucleosomes of a monocentric chromosome were clustered. The compacted holocentric chromosome displayed a line of centromeric nucleosomes, whereas the monocentric chromosome had a centromeric region with distinct compaction; contacts were insulated and LEs were highly localized in this region (Supplementary Figure S8). The exact arrangement of the chromatin fibre in the centromeric constriction is unknown, but loops in our model were smaller in this region (Figure 6, Supplementary Figure S8). The smaller loops arose from the short distance between centromeric nucleosomes, which restricted the extrusion of loops. The compaction of regions without centromeric nucleosomes resulted in larger chromatin loops (Figure 6), and the differences in loop size created a constriction-like structure.

The anchoring effect was again key to this process. The compaction is uniform along the chromosome if the LEs are not affected by the centromeric nucleosomes, which appear scattered regardless of their distribution (Figure 4). The blocking effect also produced smaller loops for the monocentromere region but could not bring the centromeric nucleosomes to a compact line and this region remained loose and very flexible (Supplementary Figure S9).

Holocentric and monocentric-like compacted chromosomes also differed in length. In line with our previous tests for centromeric nucleosomes with different interaction effects (Figure 4), the monocentric chromosome had almost twice the lenght of the holocentric chromosome (650 and 374 axial nucleosomes, respectively, Figure 6 and Supplementary Table S2). The monocentric chromosome, with a lower number of centromeric nucleosomes, resembled the setting of no effect when the chromosome length was mostly limited by the number of LEs. The holocentric chromosome, with 100 centromeric nucleosomes, resembled the setting of the anchoring effect when the chromosome length was mostly limited by the number of centromeric nucleosomes.

### The presence of a kinetochore complex might create a mitotic groove-like centromere structure in holocentrics

A longitudinal groove along each miotic sister chromatid is visible in holocentric chromosomes of *L. elegans* and *R. pubera* (10–12). We speculated that the kinetochore, composed of protein layers, restricts the LEs in space, giving a preferential direction to the emergence of chromatin loops. To test this hypothesis, we simulated the kinetochore as a large regular grid of beads next and parallelled to the centromeric nucleosomes, which were constricted to a straight line.

**Figure 7.**
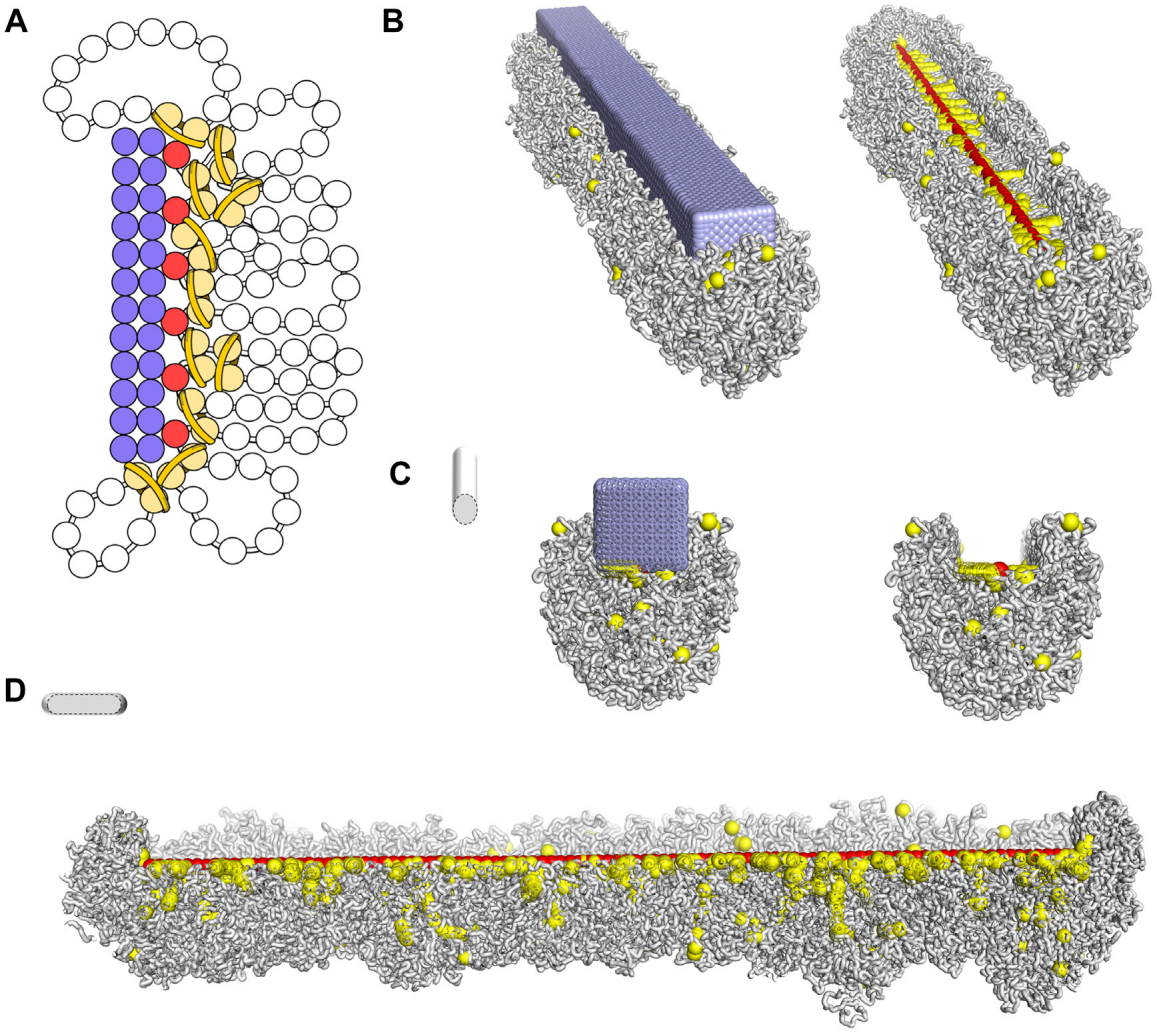
Simulation of a groove-like centromere along a holocentric condensed chromosome. (**A**) Representation of a simulated kinetochore arrangement as a rectangular bar formed by fixed spheres (in lilac). Red spheres represent the line of centromeric nucleosomes. Pairs of yellow spheres represent nucleosomes bound by LEs, and the chromatin fibre is shown as white beads on a string. (B) Final conformation of a simulated holocentric chromosome in the presence of the kinetochore arrangement. Components follow the same code color as in (A). The kinetochore is embedded in the chromatin fibre. On the right, the kinetochore is not shown so that the centromeric line is visible at the bottom of the groove and surrounded by LEs. Transversal (**C**) and longitudinal (**D**) cross-sections evidence the centromeric groove-like structure.

We observed in simulations that LEs and the emergence of loops in the three-dimensional space were restricted by the kinetochore. Lengthwise, the chromosome was restricted to the extension of the kinetochore. Radially, the chromatin fibre, organized in loops, was free to diffuse but was not able to occupy the entire space opposite to the kinetochore arrangement, thus forming a groove-like structure, and maintained the centromeric line of the chromosome (Figure 7). At the bottom of the groove, we observed associated and aligned centromeric nucleosomes and the surrounding LEs (Figure 7B,C). A transversal cut confirmed the groove-like structure (Figure 7C). Loops surrounding the longitudinal centromere created a clear contrast in the structure and covered the ends of chromosomes.

We simulated the presence of a kinetochore in the monocentric chromosome in the same way (Supplementary Figure S10). The grid of beads was set proportionally to the number of centromeric nucleosomes, reaching 400 nm in length (Supplementary Table S3). It again restricted the emergence of the loops in the centromere to one side, but did not form a groove; the chromatin loops were too small to embrace the kinetochore. Because the kinetochore attached only to a small region, the overall chromosome length (6.5 μm) was slighty reduced in this later compaction step (Supplementary Table S2).

## DISCUSSION

We propose a loop extrusion process in which centromeric nucleosomes block and anchor LEs and are brought together into a line in compacted holocentric chromosomes. This mechanistic model relies on the function and distribution of three protein complexes, broadly observed across eukaryotes, SMC proteins, CENH3-containing nucleosomes and kinetochores, which can be regulated to create variable centromeric arrangements.

SMCs, such as condensins, have been shown to act as loop extruders in the chromatin compaction process (47). Their dynamic binding and release of the chromatin fibre induce a stable condensed state of the chromosome (24). In this state, the majority of condensins are found at the axis of the chromosome, securing the basis of consecutive chromatin loops (17,36,45). Consistently in all simulations, increasing the number of LEs (which accumulate in the axis) decreased the chromatin loop length, but this may not correspond to the same degree of radial compaction, as the volume of nested loops are more complex to infer. Experimental observations for human and *C. elegans* report hundreds of condensins per 20 Mb (Supplementary Table S1).

Our simulations support that a condensed state can be achieved solely by the action of LEs, found at the axis of the chromosome, even without barriers (Figure 4B). But they showed that the alignment of the centromeric nucleosomes occurred only when they were anchored to LEs (Figure 4). About 200 LEs are enough to compact a holocentric chromosome (20 Mb), bringing the centromeric nucleosomes (100) into a line. More than 1000 of LEs seemed to cause an overload and significantly delaied the equilibration, but longer simulations could overcome this issue and reached a steady compacted state with the same chromosome length (below 400 axial nucleosomes). Without the anchoring effect, the more LEs, the longer the chromosome (Figure 4).

The motion of condensins can be affected by other proteins to approximate specific DNA sites and to form regions with distinct condensation, such as TADs containing CTCFs in interphase (48,49). Chromatin loops with restricted size also characterize heterochromatin and pericentromeric regions in mitotic chromosomes (33,50). Biologically, the anchor between centromeric nucleosomes and condensins could be mediated by the N-terminal tail of amino acid residues that CENH3 carries. Alternatively, the assembly of kinetochore proteins could trigger the anchoring at specific time points of the cell cycle. High affinity between SMC complex proteins and epigenetic factors have already been reported. For example, CTCF interacts with and anchors cohesin via a small flexible linker (40). Less than 10 amino acid residues strongly interact with residues from the SA2-SCC1 human cohesin subunits. And the epigenetic marker histone H3K4me3, together with TFIIIC, was also observed to interact with and anchor condensin II in mammals (51). So, we put forward the hypothesis that the affinity between condensins and centromeric nucleosomes may be higher than reported for canonical chromatin (52).

In our model, similar to TAD borders, adjacent centromeric nucleosomes had a high contact probability in the compacted state (Supplentary Figure S6). This proposed mechanism brings not only the centromeric nucleosomes to a linear organization but also the chromatin loops in between them as well (Figure 5). By regulating the distribution of LEs and centromeric nucleosomes, the same mechanism could form different structural arrangements and compaction levels. We observed that the modelled holocentric or monocentric-like distribution of centromeric nucleosomes led to different chromosome lengths (Figure 6 and Supplementary Table S2).

We further suggest that the presence of kinetochores in holocentric species has a direct impact on the chromosome structure (Figure 7). With a model of the kinetochore plate bound to the holocentric line, we observed a constriction to the chromatin loops, forming a groove-like structure along the chromatid. The presence of kinetochores also limited, possibly shortened, the length of the centromeric line.

With the progression of the cell cycle and breakage of the nuclear membrane, the chromosomes are exposed to the cytoplasm. This new solvation can further restrict the volume of the chromosome. But our simulations do not apply any volume constraints, and the observed shrinkage is solely because of loop compaction and assembly of kinetochores. As the compacted chromosomes adopted curved cylindrical shapes, it is hard to evaluate the volume differences, instead we analysed the compaction in terms of chromosome length.

In the first compaction step, the ubiquitous centromeric nucleosomes in holocentric chromosomes limit the chromatin loop and chromosome lengths. In a second step, the long kinetochore assembly ensures uniform compaction along the chromosome. In comparison, monocentric chromosomes fall behind in both compaction steps and are longer at the end of the simulations (Supplementary Table S2). As mitotic monocentric chromosomes have been reported with a helical coiling (36), we suggest that the coiling is an alternative compaction step not required for holocentric chromosomes.

Experimental values for spatial chromosome length also indicate some differences between monocentric and holocentric chromosomes during the cell cycle (Supplementary Table S1). When they become distinguishable, human chromosomes are longer (per 100 Mb) than *C. elegans* and *R. pubera* chromosomes, consistent with our simulations (after compaction by loops). Human chromosomes also go through higher compaction until metaphase, which might indicate a different compaction process. This short comparison, supported by our models, indicates that the spatial chromosome length might be a valuable parameter to describe chromosome organization. Recently, lengthwise compaction of mitotic chromosome was also proposed to determine the subsequent architecture in interphase (27). More data are required to confirm our observations, but we expect that future studies could relate chromosome organization to its length observed by chromosome imaging.

Despite the small size of the chromosome that we simulated, we expect that larger holocentric chromosomes are subjected to the same mechanism, but its compaction may require different amounts of LEs or result in different chromatin loop lengths. Likewise, we expect the formation of the centromeric groove-like structure to depend on the chromatin loop lengths and the arrangement of the kinetochores.

Limitations of our model lie in the simplicity of our assumptions. Here, centromeric nucleosomes can either block or not, either anchor or not the LEs. But these effects may be milder and not always block the motion of LEs (50), or only temporarily anchor the LEs. The loop extrusion mechanism lacks the possibility of LEs traversing each other (53). Also, we could infer more about the effects of centromeric nucleosomes simulating other distributions and the presence of eu- and heterochromatic regions (38). But we expect that our proposed mechanism will generally apply to centromeres and its improvements will lead to the observed variety of centromeric structures. Overall, none of the mechanisms we described is novel, but their combined action according to our dynamic model could account for the structural and evolutional diversity of the different centromere types.

## DATA AVAILABILITY

All the Fortran and Python scripts, for running and analyzing the simulations, are available in bitbucket.org/ipkdg/polymer_simulations.git.

## Supplementary Material

gkab648_Supplemental_File
