# Supplementary material for: A simple model explains the cell cycle-dependent assembly of centromeric nucleosomes in holocentric species
Source: Nucleic Acids Res. 2021 Aug 5;49(16):9053–65. doi: 10.1093/nar/gkab648 (PMC8450114; doi:10.1093/nar/gkab648)
Supplement: gkab648_Supplemental_File [file gkab648_supplemental_file.pdf]

**SUPPLEMENTARY DATA**

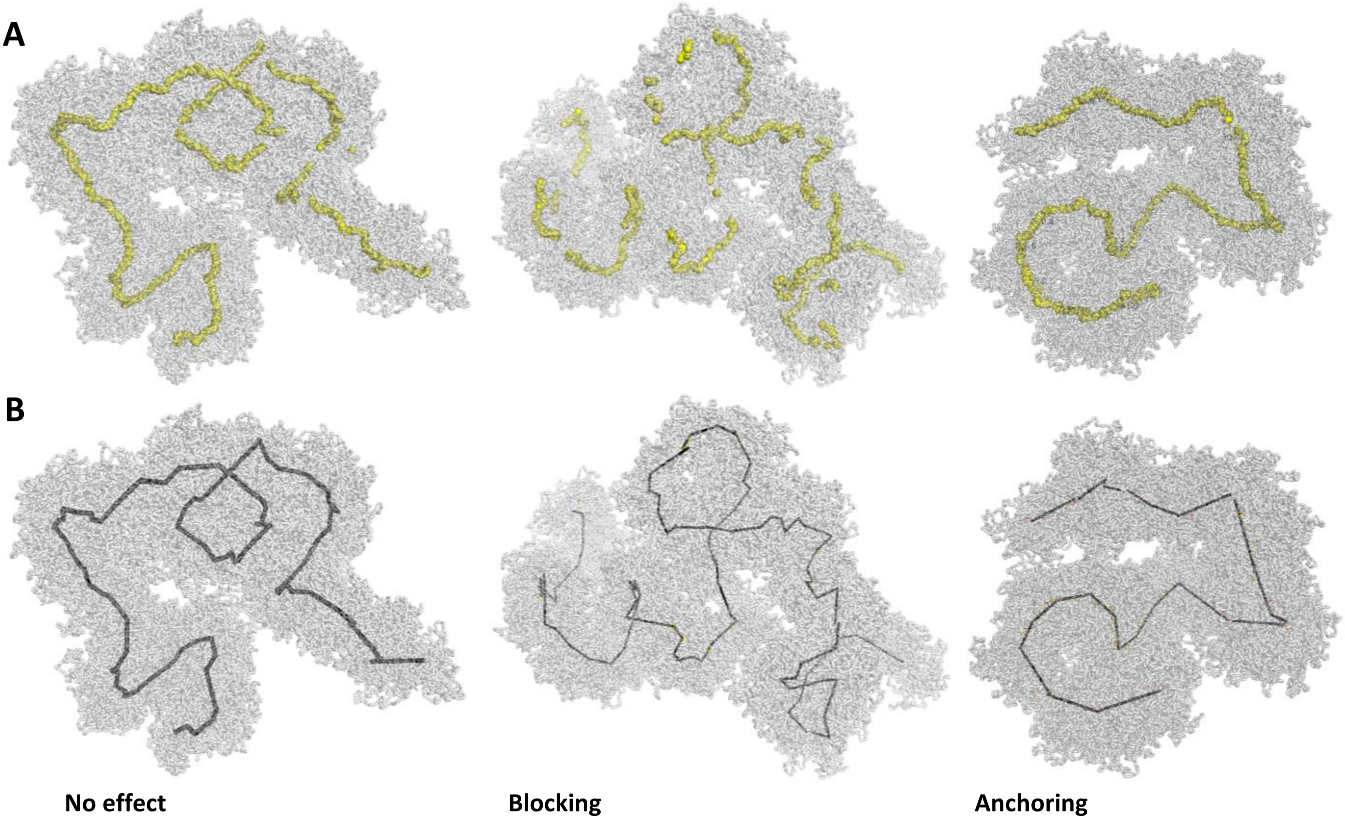


Supplementary Figure 1. (A) Distribution of axial nucleosomes (pairs of yellow beads) in the chromatin fibre (grey) in the last conformation of the simulated holocentric model with no-effect, blocking and anchoring centromeric nucleosomes, from left to right. (B) Measured segments of axial nucleosomes in the same conformations.

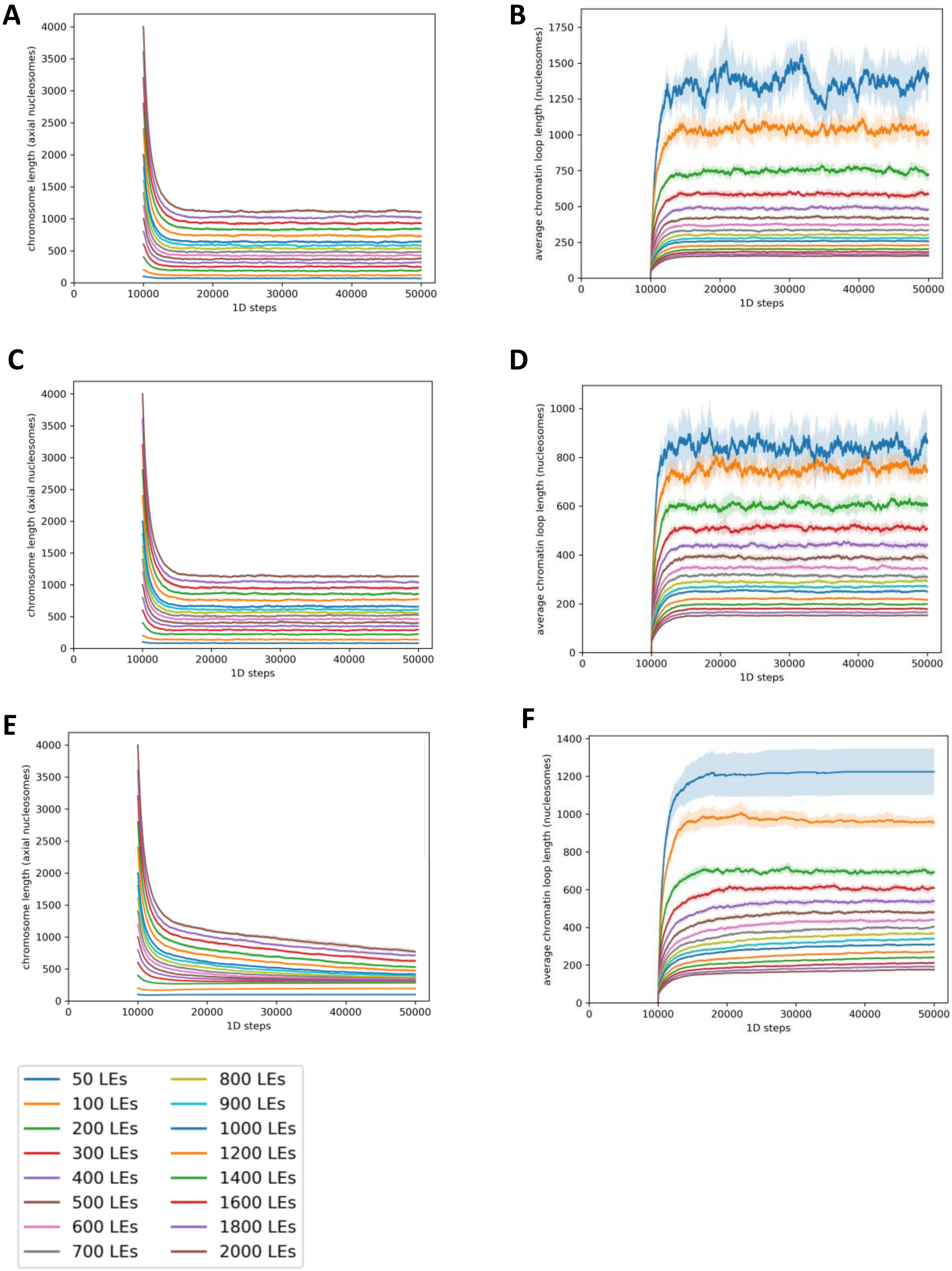


Supplementary Figure 2. Chromosome length (A, C, E) and average chromatin loop length (B, D, F) over time for different interaction effects of centromeric nucleosomes with LEs: (A, B) no effect; (C, D) blocking effect; and (E, F) anchoring effect. Simulations with different amounts of LEs are shown in different colours. A plateau for the final steps of the simulation means these two parameters have reached an equilibrium. Full lines correspond to mean values over 10 simulation replicates and the shades around them to the standard deviation.

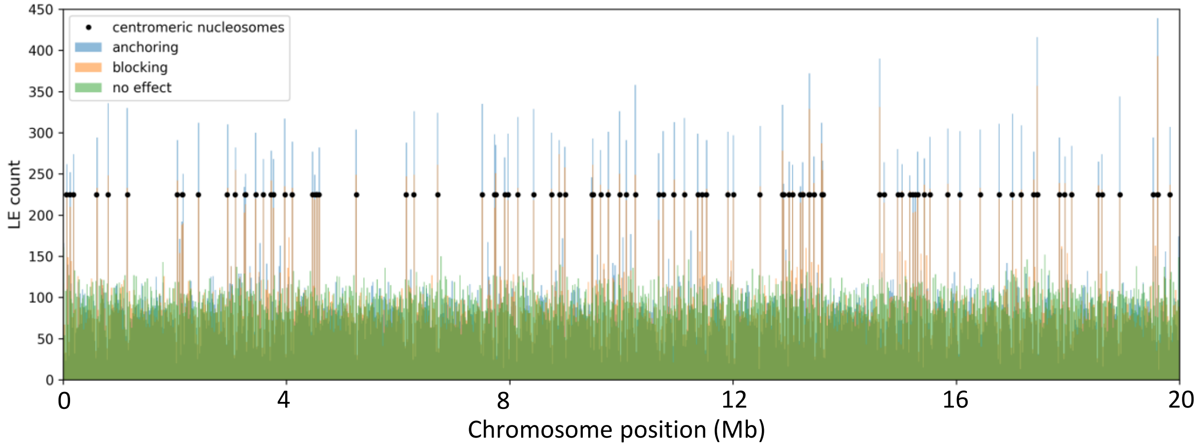


Supplementary Figure 3. Histograms of the localization of LEs along the chromosome for the three different models - no effect on, blocking or anchoring LEs. For each model we used the last conformation after 50,000 blocks of 3D steps of 50 simulations.

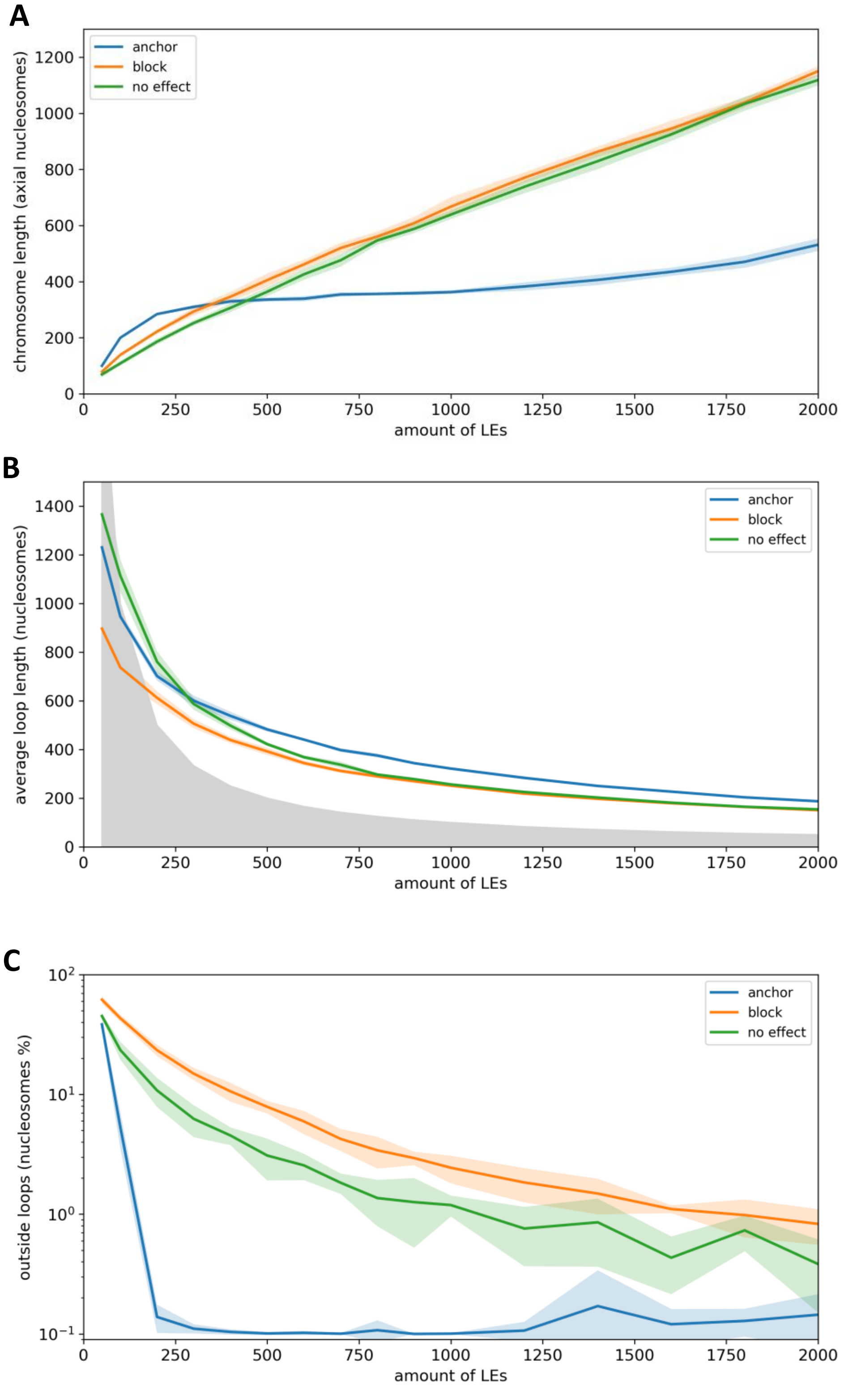


Supplementary Figure 4. (A) Chromosome length and (B) loop length calculated from 100,000 steps of 1D simulation. The grey area characterizes sparse states, when number of LEs x average loop length < 100,000 nucleosome. Above the grey area the chromosome is in a compacted state, with nested chromatin loops. (C) Percentage of nucleosomes outside chromatin loops.

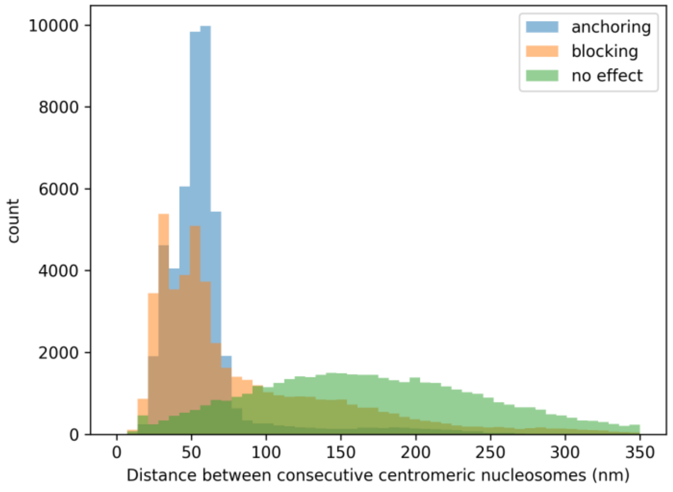


Supplementary Figure 5. Histogram of the spatial distances between centromeric nucleosomes after compaction of the holocentric chromosome for the three different models - no effect on, blocking or anchoring LEs. For each model we used 10 conformations over the last 10,000 blocks of 3D steps of 50 simulations.

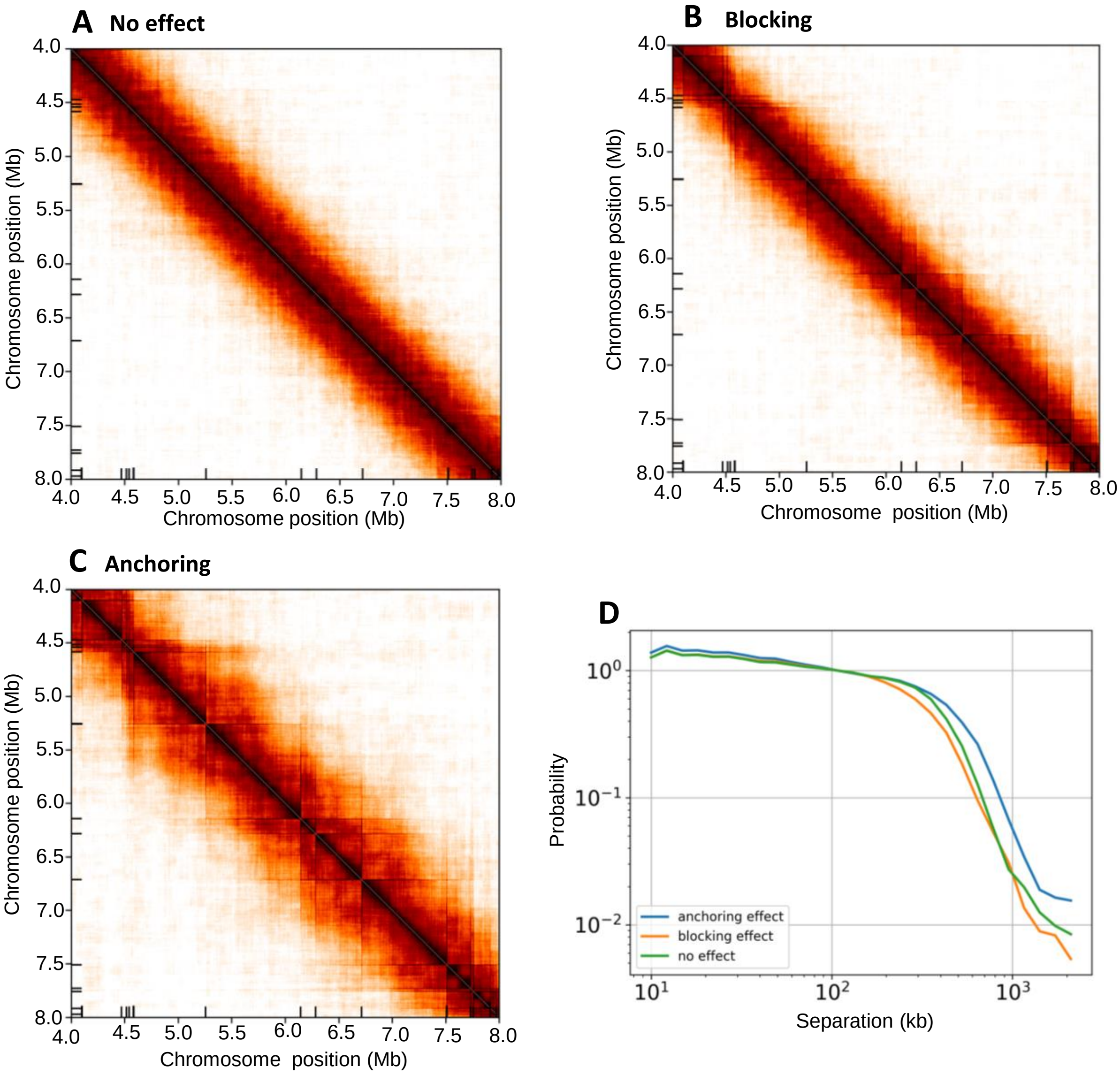


Supplementary Figure 6. (A, B, C) Contact matrices of compacted chromosomes for the three different models - no effect on, blocking or anchoring LEs. Ticks at the border mark the position of centromeric nucleosomes. (D) Contact probabilities for the three different models. The probability was normalized to 1 at 100 kb.

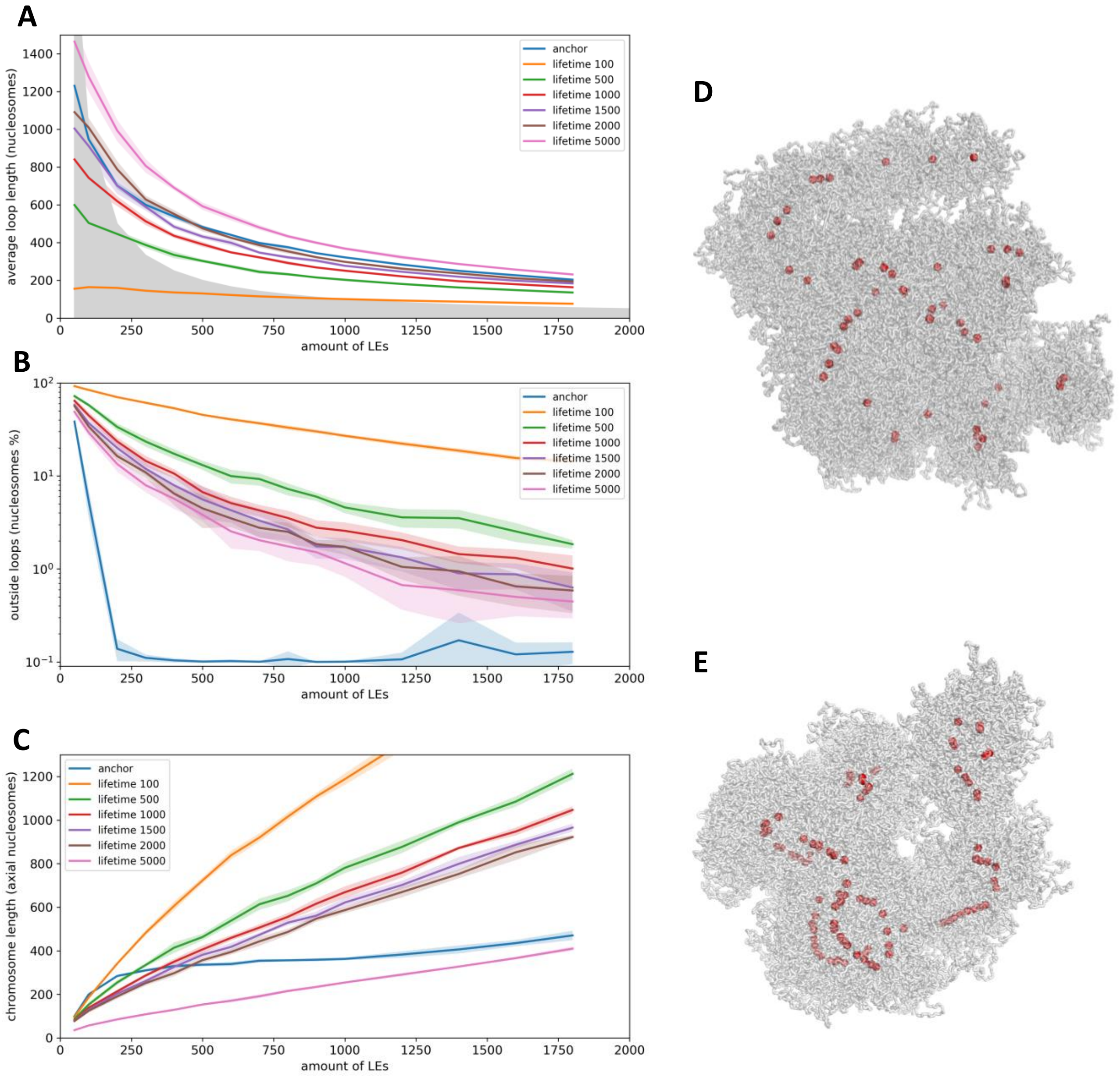


Supplementary Figure 7. Consequences of changing the lifetime in the model where centromeric nucleosomes have only a blocking effect in the loop extrusion. (A) Average chromatin loop length. (B) Percentage of nucleosomes outside chromatin loops. (C) Chromosome length. These analysis were performed with 100,000 steps of 1D simulation. (D, E) Final conformations of two 3D simulations (5,000,000 steps) of blocking effect with lifetime equal to 2,000 and 5,000, respectively.

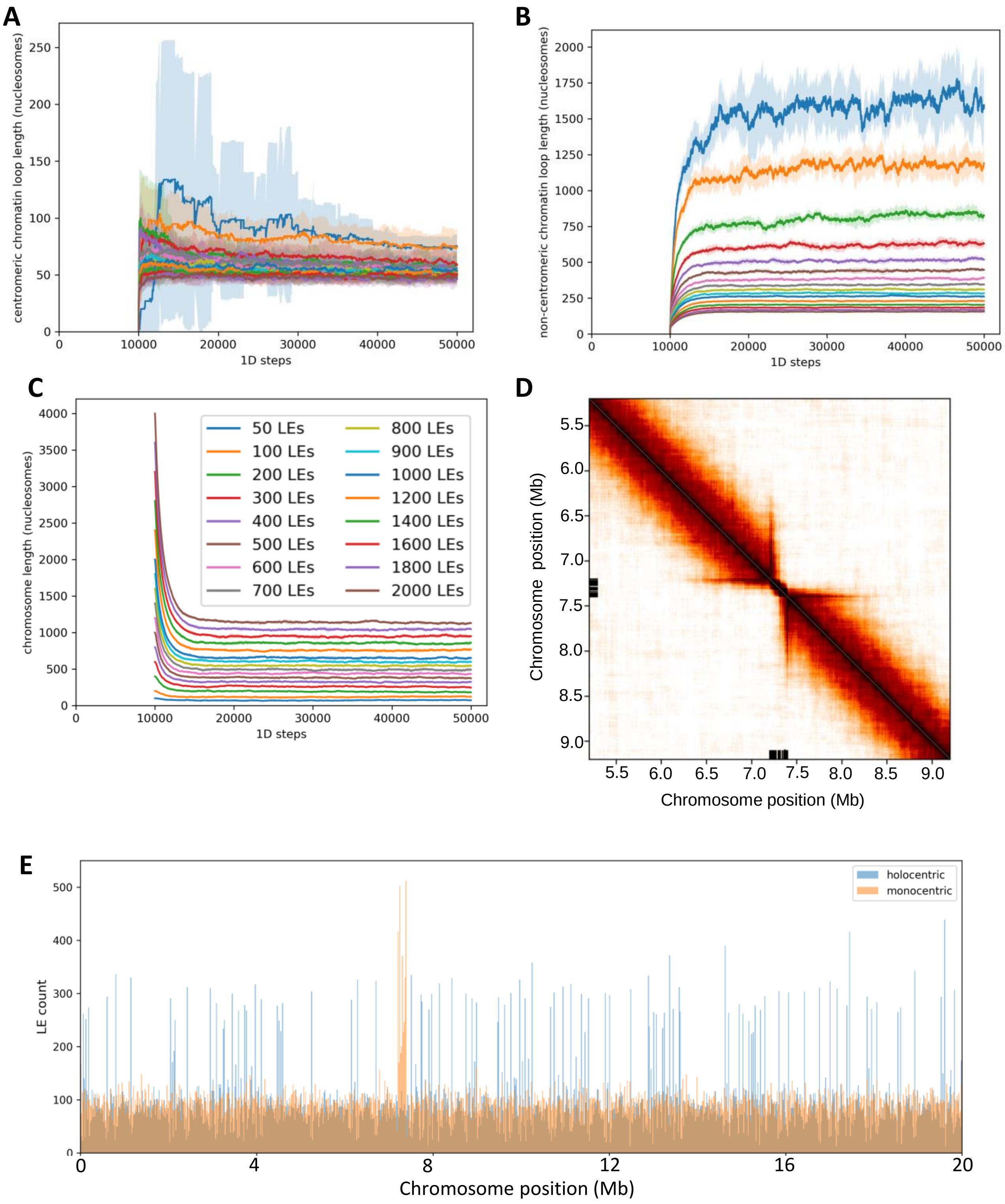


Supplementary Figure 8. Structural features of the simulated monocentric model with anchoring effect. Average chromatin loop length over time for (A) the centromeric region, and (B) outside the centromeric region. (C) Chromosome length. Simulations with different amounts of LEs are shown in different colours. A plateau for the final steps of the simulation means the length has reached equilibrium. (D) Contact matrices of the compacted monocentric chromosomes with anchoring effect and 1000 LEs. Ticks at the border mark the position of centromeric nucleosomes. (E) Histograms of the localization of LEs along the chromosome for the mono- and holocentric models. For each model we used the last conformation after 50,000 blocks of 3D steps of 50 simulations.

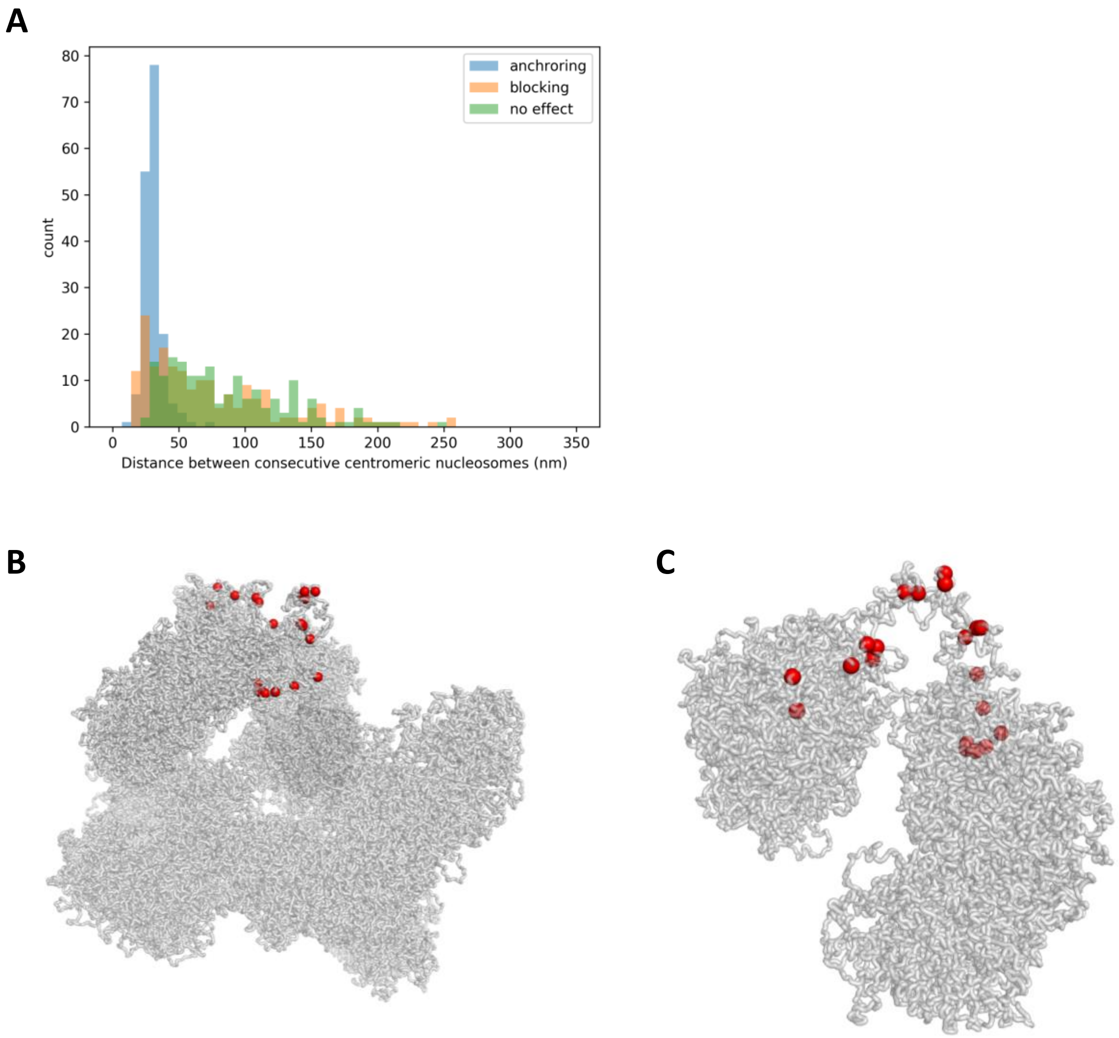


Supplementary Figure 9. Monocentric with blocking effect. (A) Histogram of the spatial distances between centromeric nucleosomes after compaction of the monocentric chromosome for the three different settings - no effect on, blocking or anchoring LEs. For each setting we used 10 conformations over the last 10,000 blocks of 3D steps of 1 simulation. (B) Last conformation of the simulated monocentric chromosome with blocking effect. (C) Zoom around the centromeric region of the same conformation.

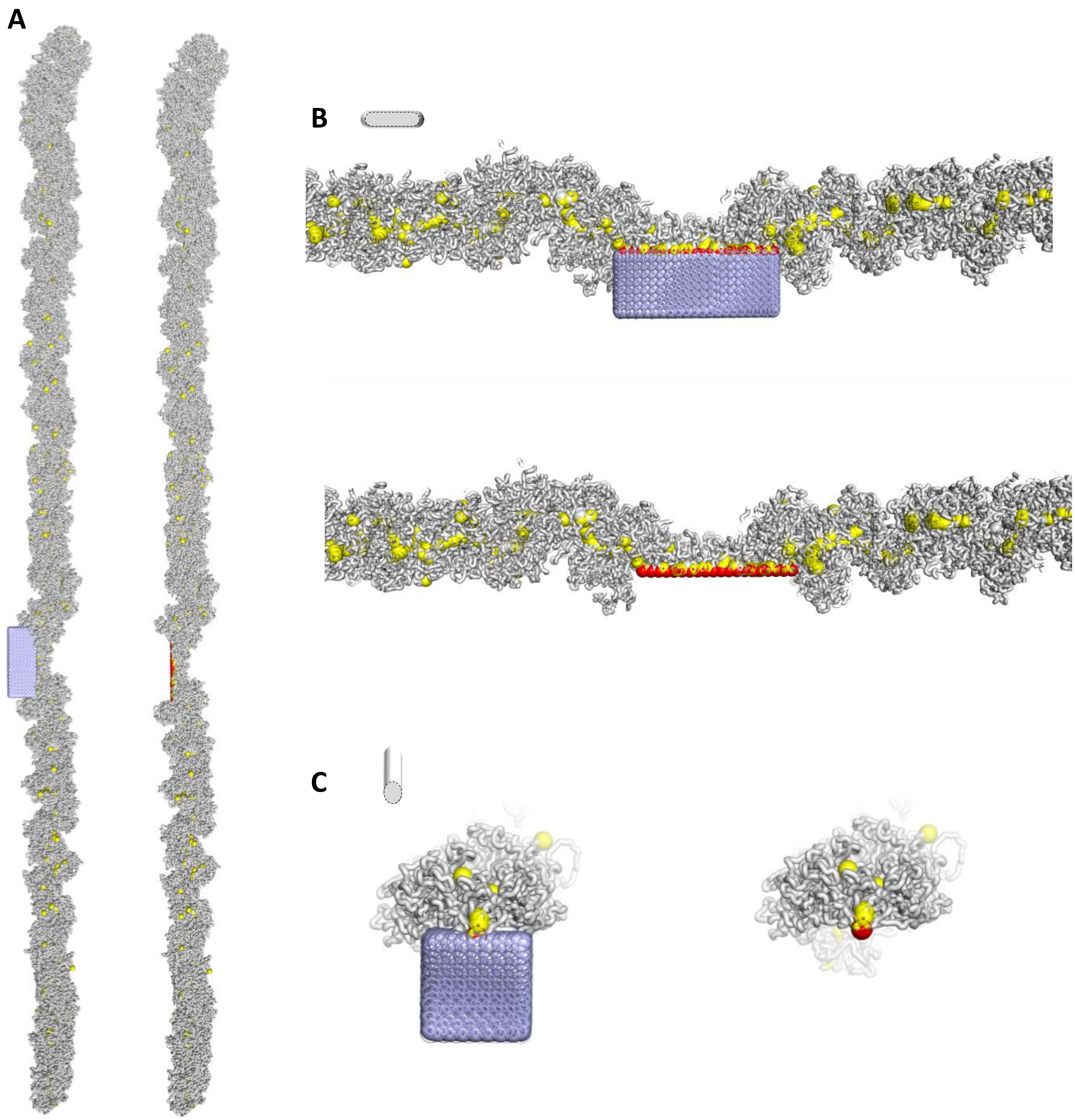


Supplementary Figure 10. Final conformation of a simulation of a monocentric chromosome, where the centromeric nucleosomes (red beads) were attached to a kinetochore grid (lilac beads). The grid is proportional to the number of centromeric nucleosomes. Pairs of yellow spheres represent nucleosomes bound by LEs and the chromatin fibre is shown in grey. A) Longitudinal view of the entire chromosome. On the right the kinetochore is not shown so that the centromeric line is visible at the side and surrounded by LEs. Longitudinal (B) and transversal (C) cross sections of the centromere region, with and without the kinetochore grid.

Supplementary Movies are currently available in the url: http://dx.doi.org/10.5447/ipk/2021/16.

Supplementary Movie 1. Condensation of a holocentric chromosome, where the centromeric nucleosomes have no effect on the Loop Extruders. Three visualization modes are present. Chromatin is shown as a 10 nm thick grey fibre, the Loop Extruders are represented by the bound nucleosomes as pairs of yellow beads and the centromeric nucleosomes are shown as beads coloured in red or in a gradient from blue to red, following their chromosome position, as indicated by the subtitles. The simulation presents 1000 LEs and 100 centromeric nucleosomes. This movie is available as HolocentricChromosome_noEffectCentromeres.mp4.

Supplementary Movie 2. Condensation of a holocentric chromosome, where the centromeric nucleosomes have a blocking effect on the Loop Extruders. Three visualization modes are present. Chromatin is shown as a 10 nm thick grey fibre, the Loop Extruders are represented by the bound nucleosomes as pairs of yellow beads and the centromeric nucleosomes are shown as beads coloured in red or in a gradient from blue to red, following their chromosome position, as indicated by the subtitles. The simulation presents 1000 LEs and 100 centromeric nucleosomes. This movie is available as HolocentricChromosome_blockingCentromeres.mp4.

Supplementary Movie 3. Condensation of a holocentric chromosome, where the centromeric nucleosomes have an anchoring effect on the Loop Extruders. Three visualization modes are present. Chromatin is shown as a 10 nm thick grey fibre, the Loop Extruders are represented by the bound nucleosomes as pairs of yellow beads and the centromeric nucleosomes are shown as beads coloured in red or in a gradient from blue to red, following their chromosome position, as indicated by the subtitles. The simulation presents 1000 LEs and 100 centromeric nucleosomes. This movie is available as HolocentricChromosome_anchoringCentromeres.mp4.

Supplementary Movie 4. Condensation of a monocentric chromosome, where the centromeric nucleosomes have an anchoring effect on the Loop Extruders. Three visualization modes are present. Chromatin is shown as a 10 nm thick grey fibre, the Loop Extruders are represented by the bound nucleosomes as pairs of yellow beads and the centromeric nucleosomes are shown as beads coloured in red or in a gradient from blue to red, following their chromosome position, as indicated by the subtitles. The simulation presents 1000 LEs and 20 centromeric nucleosomes. This movie is available as MonocentricChromosome_anchoringCentromeres.mp4.

Supplementary Table 1. Reported experimental values for the number of condensins, number of centromeric units and chromosome lengths for holocentric and monocentric chromosomes.

| | **Monocentric (human)** | **Holocentric** |
|---|---|---|
| **Condensins per Mb** | 16/Mb (1) | 28/Mb *C. elegans* (2) |
| **Centromere CENP-A** | 1/12.5 kb *(3) | 1/200 kb *C. elegans* (4) |
| **Chromosome length (base pairs)** | 47 – 247 Mb | 13.8 – 20.9 Mb *C. elegans*<br>~323 Mb *R. pubera* |
| **Chromosome length metaphase per 100 Mb** | 3.5 or 1,9 µm/100 Mb (5, 6) | ~2 µm/ 20 Mb *C. elegans* **(7)<br>~2.3 µm/100 Mb *R. pubera* **(8) |
| **Chromosome length prophase/prometaphase per 100 Mb** | 16.7µm/100 Mb (9) | ~4 µm/100 Mb *R. pubera* (8) |

* 200 CENP-A per centromere (average size 2.5 Mb)
**inspection from published images and personal communication

Supplementary Table 2. Observed chromosome length after 1D and 3D simulations as function of nucleosomes or nm for four different simulated models, at their compacted final conformation.

| **Chromosome type** | **Holocentric** | | | **Monocentric** |
|---|---|---|---|---|
| **Centromeric nucleosome effect** | no effect | blocking | anchoring | anchoring |
| **1D simulation** | | | | |
| **Chromosome length (nucleosomes)** | 624 | 648 | 374 | 650 |
| **Compacted 3D model** | | | | |
| **Nucleosomes median distance (nm) / peak distance (nm)** | 13.4 /12.5 | 13.3 / 12.5 | 14.5 / 12.5 | 13.5 / 13.0 |
| **Calculated chromosome length (nm)** | 8,362 / 7,800 | 8,618 / 8,100 | 5,423 / 4,675 | 8,775 / 8,450 |
| **Measured chromosome length (nm)** | 7,989 | 9,394 | 4,995 | 7,884 |
| **3D model with kinetochore** | | | | |
| **Chromosome length (nucleosomes)** | | | 362 | 636 |
| **Nucleosomes average distance (nm) / peak distance (nm)** | - | - | 12.3 / 10.5 | 13.5 / 12.0 |
| **Calculated chromosome length (nm)** | - | - | 4,453 / 3,801 | 8,586 / 7,632 |
| **Measured chromosome length (nm)** | - | - | 2,300 | 6,500 |

Supplementary Table 3. Summary of simulation parameters along with their justifications.

| Parameter | Used values | Justification |
|---|---|---|
| ***Speed of Loop Extruders*** | 2 nucleosomes/ 100 steps | Short period between LE steps with low energy cost. |
| ***Lifetime*** | 100,000 steps | Time required for a LE to span twice the average distance between two centromeric nucleosomes in the holocentric distribution. |
| ***Number and distribution of centromeric nucleosomes*** | Holocentric - 100 centromeric nucleosomes/ 100,000 nucleosomes (20 Mb)<br>Monocentric – 20 centromeric nucleosomes/ 2,000 nucleosomes (400 kb) | Similar to experiments (Supplementary table 1). |
| ***Kinetochore shape*** | Holocentric - 45,000 beads forming a regular grid of 150 x 150 x 2000 $nm^3$<br>Monocentric - 9,000 beads forming a regular grid of 150 x 150 x 400 $nm^3$ | The 2 μm length matches the real length of mitotic *C. elegans* (7) chromosomes.<br>The width mimics the groove observed in *L. elegans* and *R. pubera* (8, 10). |

**References**


1. Walther,N., Hossain,M.J., Politi,A.Z., Koch,B., Kueblbeck,M., Ødegård-Fougner,Ø., Lampe,M. and Ellenberg,J. (2018) A quantitative map of human Condensins provides new insights into mitotic chromosome architectureA quantitative 3D map of Condensins in mitosis. *J Cell Biology*, **217**, 2309–2328.

2. Kranz,A.-L., Jiao,C.-Y., Winterkorn,L.H., Albritton,S.E., Kramer,M. and Ercan,S. (2013) Genome-wide analysis of condensin binding in Caenorhabditis elegans. *Genome Biol*, **14**, R112.

3. Bodor,D.L., Mata,J.F., Sergeev,M., David,A.F., Salimian,K.J., Panchenko,T., Cleveland,D.W., Black,B.E., Shah,J.V. and Jansen,L.E. (2014) The quantitative architecture of centromeric chromatin. *Elife*, **3**, e02137.

4. Steiner,F.A. and Henikoff,S. (2014) Holocentromeres are dispersed point centromeres localized at transcription factor hotspots. *Elife*, **3**, e02025.

5. Yunis,J.J., Sawyer,J.R. and Ball,D.W. (1978) The characterization of high-resolution G-banded chromosomes of man. *Chromosoma*, **67**, 293–307.

6. Bender,M.A. and Kastenbaum,M.A. (1969) Statistical analysis of the normal human karyotype. *Am J Hum Genet*, **21**, 322–51.

7. Maddox,P.S., Portier,N., Desai,A. and Oegema,K. (2006) Molecular analysis of mitotic chromosome condensation using a quantitative time-resolved fluorescence microscopy assay. *Proc National Acad Sci*, **103**, 15097–15102.

8. Marques,A., Ribeiro,T., Neumann,P., Macas,J., Novák,P., Schubert,V., Pellino,M., Fuchs,J., Ma,W., Kuhlmann,M., *et al.* (2015) Holocentromeres in Rhynchospora are associated with genome-wide centromere-specific repeat arrays interspersed among euchromatin. *Proc National Acad Sci*, **112**, 13633–13638.

9. Yunis,J.J. (1981) Mid-prophase human chromosomes. The attainment of 2000 bands. *Hum Genet*, **56**, 293–298.

10. Heckmann,S., Schroeder-Reiter,E., Kumke,K., Ma,L., Nagaki,K., Murata,M., Wanner,G. and Houben,A. (2011) Holocentric Chromosomes of *Luzula elegans* Are Characterized by a Longitudinal Centromere Groove, Chromosome Bending, and a Terminal Nucleolus Organizer Region. *Cytogenet Genome Res*, **134**, 220–228.
